# Carbon monoxide-oxidising Pseudomonadota on volcanic deposits

**DOI:** 10.1186/s40793-025-00672-y

**Published:** 2025-01-26

**Authors:** Robin A. Dawson, Nicola Fantom, Tamara Martin-Pozas, Patricia Aguila, Gary M. King, Marcela Hernández

**Affiliations:** 1 School of Biological Sciences, University of East Anglia, Norwich, NR4 7TJ UK; 2 Department of Biology and Geology, University of Almería, 04120 Almería, Spain; 3 Laboratorio de Microbiología Molecular, Escuela de Tecnología Médica, Universidad Austral de Chile, Juan Soler Manfredini, 1771 Puerto Montt, Chile; 4 Department of Biological Sciences, Louisiana State University, Baton Rouge, LA 70803 USA

**Keywords:** Carbon monoxide, Volcanic deposits, *Cupriavidus*, *Paraburkholderia*, Carbon monoxide dehydrogenase

## Abstract

Carbon monoxide (CO) oxidising microorganisms are present in volcanic deposits throughout succession, with levels of vegetation and soil influencing the communities present. Carboxydovores are a subset of CO oxidisers that use CO as an energy source, which raises questions about the physiological and metabolic features that make them more competitive in harsh volcanic ecosystems. To address these questions, samples were taken from volcanic strata formed by eruptions from Calbuco Volcano (Chile) in 2015 (tephra) and 1917 (soil). Two carboxydovore members of the Burkholderiaceae family were isolated for further study to elucidate the benefits of carboxydovory for the survival of these strains in extreme volcanic ecosystems. The isolates were identified as *Paraburkholderia terrae* COX (isolated from the 2015 tephra) and *Cupriavidus str. CV2* (isolated from the 1917 soil). 16S rRNA gene sequencing showed that within the family Burkholderiacea, the genus *Paraburkholderia* dominated the 2015 volcanic deposit with an average relative abundance of 73.81%, whereas in the 1917 volcanic deposit, *Cupriavidus* accounted for 33.64% (average relative abundance). Both strains oxidise CO across a broad range of concentrations (< 100 ppmv – 10,000 ppmv), and genome sequence analysis revealed a candidate form-I carbon monoxide dehydrogenase (CODH), which is likely to catalyse this process. Each strain oxidised CO specifically at stationary phase but the conditions for induction of CODH expression were distinct. *Cupriavidus* strain CV2 expressed CODH only when CO was added to cultures (100 ppm), while *Pb. terrae* COX expressed CODH regardless of supplementary CO addition. Based on comparative metabolic and phylogenetic analyses, *Cupriavidus* strain CV2 is proposed as a novel species within the genus *Cupriavidus* with the name *Cupriavidus ulmosensis* sp. nov. for the type strain CV2^T^ (= NCIMB 15506^ T^, = CECT 30956^ T^). This study provides valuable insights into the physiology and metabolism of carboxydovores which colonise volcanic ecosystems.

**Supplementary Information:**

The online version contains supplementary material available at 10.1186/s40793-025-00672-y.

## Introduction

Soils act as a significant sink for CO due to microbial activity, accounting for removal of ~ 250–300 Tg yr^−1^ [1, 2]. Both aerobic and anaerobic microorganisms are capable of CO oxidation due to the use of a Mo–Cu or Ni–Fe carbon monoxide dehydrogenases (CODH), respectively [3, 4], although limited evidence is available to suggest that anaerobic CO oxidation plays a role in atmospheric CO consumption. CO oxidisers colonise diverse ecological niches, and isolates have been retrieved from terrestrial, aquatic, and even clinical samples [2, 5, 6]. CO oxidisers can be broadly classified into two groups according to the specific usage of CO; carboxydotrophs and carboxydovores. All known oxygen-tolerant CO-oxidising bacteria use Mo–Cu carbon monoxide dehydrogenase (CODH) to oxidise CO to CO_2_, yielding energy. *coxMSL* encodes the medium (FAD-binding), small (2 x [2Fe-2S]) and large (Mo–Cu) subunits of CODH, respectively, with CoxL containing the active site and critical molybdopterin cytosine dinucleotide cofactor [7, 8]. Carboxydotrophs can grow using CO as the sole source of carbon and energy, with many isolates able to use high concentrations of CO (e.g. 10% (v/v) [2]. CODH catalyses the oxidation of CO to CO_2_, meaning that carboxydotrophs use pathways such as the Calvin-Benson-Basham cycle to fix the generated CO_2_ while also gaining ATP and NAD(P)H + H^+^ [9]. Conversely, while carboxydovores may oxidise high concentrations of CO they can also oxidise atmospheric levels but only benefit from this reaction by acquiring energy in the form of electrons coupled to ATP generation [1, 3].

The physiology, biochemistry, and genetics of carboxydotrophs have been studied in detail, with many studies focussing on the model bacterium *Afipia carboxidovorans* OM5^T^ (formerly *Oligotropha carboxidovorans* OM5^T^) [9–12]. However, relatively few publications focus on bacterial carboxydovores in similar detail [1, 3, 5, 13]. Representatives from both groups use diverse metabolic strategies, as a common observation in carboxydovores is a switch from heterotrophy to CO oxidation as the cell begins to starve [1, 13, 14]. Conversely, the carboxydotroph *Hydrogenophaga pseudoflava* ceases assimilation of carbon derived from CO oxidation when growing in the presence of organic carbon, instead using the energy from CO oxidation to enhance anabolism of carbon from heterotrophy [15]. Differing from both above examples, the carboxydotroph *Mycobacterium* sp. JC1 employs a mixotrophic approach where CO oxidation was detected in crude cell extracts during chemoautotrophic growth with CO and during heterotrophic growth while CO was present [16]. These examples highlight the metabolic versatility of CO-oxidisers from both groups, demonstrating that carboxydovores and carboxydotrophs are more complex in their lifestyle and metabolism than can be accounted for by the presence or absence of growth with CO as the sole carbon and energy source.

Trace gas oxidation is important during soil formation and development to fulfil the energy requirements of resident bacteria [17]. These processes account for 2–10% of reducing equivalent flow in young volcanic deposits from Kilauea Volcano [18], suggesting that the use of trace gases is a significant contributor to microbial life in such environments. As soils age and plant life develops, available organic carbon increases and the contributions of CO oxidation to microbial metabolism become less important [18]. Although, CO oxidation may continue to be supported through both atmospheric and biological sources [19]. Fresh volcanic deposits are harsh ecosystems, which present many challenges to microbial colonisers. pH varies spatially, in part due to acidification by volcanic ash [20], and organic matter is very limited but may be augmented through processes such as chemoautotrophy and chemolithotrophy [21, 22], necromass or allochthonous carbon deposition [23]. Additionally, without layers of soil, fresh deposits are not buffered against fluctuating temperature and potential desiccation. Such factors present significant challenges for microbial colonisation.

Recent studies have begun to demonstrate that carboxydovores are abundant and environmentally important [24], especially as this group is able to oxidise atmospheric CO [13]. Therefore, the aim of this study was to address the lack of cultured carboxydovore isolates by performing targeted enrichment and isolation of carboxydovore bacteria from volcanic deposits. This work also sought to advance our understanding of the physiological and metabolic strategies employed by CO-oxidising bacteria colonising these environments. A modified isolation method was developed to target bacteria that use low concentrations of CO as a supplementary energy source, leading to the isolation of two novel carboxydovore species within the Pseudomonadota (formerly Proteobacteria). Physiological, biochemical, and genomic analyses revealed their metabolic flexibility and unique adaptation to carbon limitation through CO utilisation. Furthermore, to better understand the relevance of these strains in the environmental samples from a microbial ecology perspective, 16S rRNA gene and functional gene (*coxL*) sequencing were employed to examine the diversity of the total microbial community and specifically CO oxidisers in volcanic deposits of different ages at Calbuco Volcano, Chile.

## Materials and methods

### Collection and chemical analysis of volcanic soils

Soil samples were collected in February 2022 from Calbuco Volcano (41.3304° S, 72.6087° W), Chile. Samples were taken from a tephra stratification (mostly ash, Fig S1) formed by eruptions in 1893, 1917, 1961 and 2015 (the most recent eruption), characterised by different levels of soil development. Physical details of the tephra stratification have been reported previously [25]. This site was used for grazing cattle circa 1940–1998 (Barbara Corrales, Parque Volcánico Valle Los Ulmos, Chile, *pers. comm*). Soil samples were collected in triplicate at a 30 cm depth horizontally using a shovel, between each tephra layer, into the vertical surface of the tephra strata (Figure S1) except for the topmost layer, which consisted only of volcanic rocks. Vegetation was removed before sampling. In the youngest deposit (2015), approximately 5 cm of volcanic rocks were removed before sampling to a depth that did not extend into the horizon of the 1961 stratum. The surface of the 2015 deposit was composed almost entirely of bare rock, primary basaltic andesite tephra [25], with very little soil observed. In contrast, the layers formed by preceding eruptions consisted of soil with little to no vegetation (Figure S1). Samples (~ 0.5 kg each) were stored in polyethylene bags (Ziploc bags) and transported immediately after sampling campaign to a laboratory in Chile, where they were refrigerated at 4 °C for approximately three months before being sent to our laboratory in the UK where they continued to be stored at 4 °C for a further 4 months prior to initial enrichments. The isolates discussed in this study were isolated from the tephra layers formed by eruptions in 2015 and 1917. Physico-chemical properties were measured by “Laboratorio de analysis de suelos y plantas”, Universidad de Concepción, Chile (*see additional methods in the SI for more details*, Table S1).

### Targeted enrichment and isolation of carboxydovores from volcanic soil

Slurries were prepared by mixing volcanic soils with VL55 medium (pH 5.5, DSMZ recipe 1266) at a ratio of 1 g:1 ml, supplemented with 1 µl ml^−1^ vitamin solution (DSMZ recipe 1266). Media components were purchased from Sigma-Aldrich, with the exception of MES hydrate (Formedium). Carbon sources were ~ 100 ppmv CO (CK Isotopes, UK) and 0.5 mM pyruvate to support mixotrophy (supplied as sodium pyruvate (Formedium)). The slurries were incubated at 30 °C with shaking at 100 rpm (Infors HT Ecotron). Headspace CO was measured daily (see Sect. “Measurement of headspace CO”) until it became undetectable, then the headspace was amended with approximately 100–200 ppmv CO. Following the consumption of three additions of CO (576 h for 2015 tephra enrichments, 150 h for 1917 soil enrichments), 1 ml of the enrichment medium (without soil matter) was transferred to 11 ml of fresh VL55 with 0.5 mM pyruvate and ~ 100 ppmv CO. Headspace analysis and amendments with CO were conducted under the same incubation conditions as above until a further 300 ppmv CO was consumed (500 h). The enrichment medium was diluted across a tenfold series to 10^–5^ and 100 µl of diluted enrichment medium was spread on solid VL55, set using 1.5% (w/v) Bacto agar (Fisher Scientific) and amended with 0.5 mM pyruvate. Plates were incubated at 30 °C (Sanyo MIR-153) until colonies formed over 7–10 days.

Colonies were screened for the presence of form-I *coxL*, the active site-containing component of CODH, using primers OMPf (5’-GGCGGCTT[C/T]GG[C/G]AA[C/G]AAGGT-3’) and O/BR (5’-[C/T]TCGA[T/C]GATCATCGG[A/G]TTGA-3’) [3]. The PCR protocol was conducted as described previously [3] with modifications to use colony biomass as the template. 5% (w/v) DMSO and 0.23% (w/v) BSA were added to the DreamTaq PCR mastermix (ThermoScientific, Waltham, MA) and colony biomass was transferred directly to the PCR reaction mixture using a sterile pipette tip. The initial denaturation was maintained at 94 °C for 10 min in a G-Storm GS0002M thermal cycler (Labtech) to lyse cells and release the DNA template. Amplification cycles proceeded as follows: denaturation 94 °C – 45 s, touchdown annealing (62 °C initial annealing, decreasing by 1 °C per cycle to a final value of 58 °C, which was held for 30 cycles) – 60 s, then extension 72 °C – 90 s. Final extension was held for 20 min at 72 °C. PCR products were visualised using 1% (w/v) agarose gel electrophoresis, post-stained using 1 µl ml^−1^ GelRed Nucleic Acid Gel Stain (Biotium). Colonies that contained form-I *coxL* were re-inoculated in sterile VL55 medium with 2 mM pyruvate and 100 ppmv CO and incubated at 30 °C for up to 2 weeks with shaking at 100 rpm. Cultures that consumed CO were serially diluted across a tenfold series to 10^–5^ and spread on solid VL55 agar with 2 mM pyruvate. Colony morphology was examined for evidence of contamination and colonies were re-tested for the presence of form-I *coxL*. This process was repeated until pure cultures were obtained (2 × each). Two isolates were further characterised in this study and named *Cupriavidus ulmosensis* CV2^T^ and *Paraburkholderia terrae* COX.

### Cryo-scanning electron microscopy

All preparation steps were performed within the cryo-scanning electron microscopy (cryo-SEM) preparation system PP30010T (Quorum Technologies). 1 mm blocks of VL55 agar were fitted on grooved stubs and dipped in nitrogen slush under vacuum. Samples were then transferred (still under vacuum) into the preparation chamber and sublimated at − 90 °C for 3 min before being sputter-coated with platinum. The samples were subsequently transferred into the SEM (Gemini-SEM 300, Zeiss GmbH). Images were acquired at 2 kV acceleration voltage using the secondary electron detector.

### Cultivation of aerobic carboxydovores

The growth substrate ranges of each strain were tested by adding 5 mM of a carbon source to VL55 medium (pH 5.5) as the sole source of carbon and energy. 1 M carbon source stock solutions (Table S2) were prepared in distilled water and sterilised by passage through 0.2 µm syringe filters, then added to a final concentration of 2–5 mM in VL55 medium according to experimental requirements. All pure cultures were maintained at 30 °C with shaking at 150 rpm (Infors HT, Ecotron). Culture densities were recorded at 600 nm using a UV-1800 spectrophotometer (Shimadzu, UK) with two biological replicates. The pH range of VL55 medium was buffered to pH 4.0 using citric acid-Na_2_HPO_4_ buffer (0.1 M and 0.2 M, respectively), pH 5.0–6.0 using MES (2-(N-morpholino)ethanesulfonic acid), and pH 7.0–8.0 using Trizma base. pH was adjusted using HCl or NaOH. pH tolerance experiments were conducted using 5 mM pyruvate to support growth at 30 °C in 20 ml final volume VL55 medium until stationary phase (48 h), using three biological replicates. Temperature and salinity tolerance experiments were conducted in 20 ml final volume VL55 medium using 5 mM pyruvate. Cultures were maintained until stationary phase (48–72 h) at 25 °C, 30 °C, 37 °C or 45 °C with shaking at 150 rpm (Infors HT, Ecotron) (*n* = 3). Salinity tolerance experiments were conducted at 30 °C using VL55 amended with 1% or 10% (w/v) NaCl (Formedium) (*n* = 3). To confirm that our isolates are carboxydovores (i.e. unable to grow using CO as the sole source of carbon and energy), each strain was initially grown using 5 mM pyruvate and 100 ppmv CO to induce CODH expression (see below). CO was added to the headspace of 120 ml vials, sealed using butyl rubber stoppers and aluminium crimp caps, from a 5,000 ppmv stock prepared in N_2_. Cultures were transferred into fresh VL55 medium with 1% (v/v) or 10% (v/v) CO as the sole carbon source, using three biological replicates per condition, then incubated for 17 days.

### Measurement of headspace CO

CO was measured by injecting 100 µl of headspace gas into an Agilent 7890A gas chromatograph fitted with an Agilent HP-Molsieve PLOT (Porous Layer Open Tubular) column (30 m length, 0.53 mm bore, 25 µm film, 7 inch cage) at an initial oven temperature of 50 °C, programmed at a rate of 10 °C/min to 100 °C with no initial hold time, injector at 250 °C (1:2 split ratio) and flame ionization detector at 300 °C (carrier gas He, 4 ml min^−1^). Headspace CO was quantified relative to standards (CK isotopes, UK) containing a known quantity of CO (1 ppmv – 11,000 ppmv) prepared in N_2_. The limit of detection of this GC apparatus for CO is approximately 500 ppbv.

### Oxidation of elevated CO concentrations by carboxydovores

*C. ulmosensis* CV2^T^ and *Pb. terrae* COX were inoculated in VL55 medium with 5 mM pyruvate to support growth. The headspace of 120 ml vials was amended with 100 ppmv, 200 ppmv, 1,000 ppmv, 10,000 ppmv (1% v/v) or 100,000 ppmv (10% v/v) CO, using three biological replicates per condition. Headspace CO concentrations were measured by gas chromatography (as described in Sect. “Measurement of headspace CO”) for a maximum of 14 days, or until all detectable CO was consumed. Culture density (OD_600_) was measured using a spectrophotometer every 24 h until growth ceased.

### Differential CO uptake activity under different growth conditions and stages of growth

The isolates were cultivated in 400 ml VL55 medium in 2 L flasks with 5 mM pyruvate ± 200 ppmv CO, using three biological replicates per condition. 10 ml aliquots of cultures were harvested at exponential phase (18–24 h) or stationary phase (72 h) by centrifuging at 4,000 g for 10 min at 4 °C, followed by resuspension to an OD_600_ of 4.0 in 1 ml of fresh VL55 medium. Culture densities (OD_600_) were determined by tenfold dilution in sterile VL55 medium before measurement in a UV-1800 spectrophotometer (Shimadzu, UK). Cell suspensions were kept on ice in sealed 30 ml vials during transfer to a 30 °C water bath with shaking at 150 rpm. Vials were allowed to pre-warm for 3 min, then 200 ppmv CO was added to the headspace from a 5,000 ppmv stock of CO in N_2_. After a further 1 min, a headspace sample was measured by gas chromatography (Sect. “Measurement of headspace CO”). Headspace samples were measured every 7.5 min until six samples had been taken. Abiotic VL55 medium and heat-killed cell controls were tested under the same conditions. Additionally, the effect of pH on CO uptake was tested by growing *C. ulmosensis* CV2^T^ and *Pb. terrae* COX in VL55 medium buffered to pH 5.0, 6.0, 7.0, or 8.0, depending on pH tolerance. Cells were harvested at stationary phase and the rate of CO consumption was measured (as above).

### Genome sequencing and annotation

DNA was extracted from late-exponential phase bacterial cultures (OD_600_ = 0.6) grown in VL55 medium with 5 mM pyruvate using the Qiagen Genomic-tip 100/G DNA isolation kit (Qiagen) according to the manufacturer’s instructions. Genomic DNA concentration was quantified using a Qubit dsDNA HS Assay Kit (Thermo Fisher Scientific).

The strains were initially identified by amplifying the 16S rRNA gene using primers 27F/1492R [26], and Sanger sequencing was conducted by Eurofins Genomics (UK). Subsequently, whole genome sequencing was performed by MicrobesNG (Birmingham, UK). Genomic DNA libraries were prepared using the Nextera XT Library Prep Kit (Illumina, San Diego, USA) following the manufacturer’s protocol with the following modifications: input DNA was increased twofold, and PCR elongation time was increased to 45 s. DNA quantification and library preparation were carried out on a Hamilton Microlab STAR automated liquid handling system (Hamilton Bonaduz AG, Switzerland). Libraries were sequenced on an lllumina NovaSeq 6000 (Illumina, San Diego, USA) using a 250 bp paired end protocol. Reads were trimmed using Trimmomatic version 0.30 [27] and the quality was assessed using in-house scripts combined with the following software: Samtools [28], BedTools [29] and bwa-mem [30]. De novo assembly was performed using SPAdes version 3.7 [31], and contigs were annotated using Prokka 1.11 [32]. The assembly metrics were calculated using QUAST (v5.0.2) [33]. Completion and contamination metrics for the two isolates were performed using CheckM program [34]. Identification was done using the software Kraken [35] and Sina (v1.6.0) [36] using Silva 16S rRNA gene sequence database (release 128). Genome sequence annotation of both isolates were performed using MicroScope (https://mage.genoscope.cns.fr/microscope (accessed 05/06/2023)), an online platform by GenoScope (France) providing a collection of bioinformatic tools for annotation. Médigue et al. [37] has described all the software and databases integrated in the MicroScope pipelines used to perform the genome annotation.

The form-I CODH-encoding genes (*coxMSL*) from *A. carboxidovorans* OM5^T^ [38] were used as queries in tBLASTn analysis against our genomes to identify putative *cox* gene clusters. The translated CoxL component was aligned against a database of corresponding CoxL sequences using MEGA11 [39] to confirm the presence of the form-I CoxL active site motif (AYXCSFR) [21]. The genomes were uploaded to the Rapid Annotation using Subsystem Technology (RAST—https://rast.nmpdr.org/rast.cgi) server for subsystems analysis. As *Pb. terrae* COX lacked cell division and cell cycle-related genes (according to RAST analysis), this genome was uploaded to BLASTKOALA (https://www.kegg.jp/blastkoala/) for KEGG mapping using default parameters.

To determine whether our two isolates were members of previously undescribed species, the genome sequence data were uploaded to TYGS (https://tygs.dsmz.de) for whole-genome based taxonomic analysis and for *d*DDH (digital DNA-DNA hybridisation) computed with GGDC (Genome-to-Genome Distance Calculator) [40]. Information on nomenclature, synonymy and associated taxonomic literature was provided by TYGS’s sister database, the List of Prokaryotic names with Standing in Nomenclature (LPSN, available at https://lpsn.dsmz.de) [41]. Additionally, using Microbial Genomes Atlas Online (MIGA [42]), average nucleotide identity (ANI) was calculated. Finally, average amino acid sequence identity (AAI) was calculated using the BLAST tool with aai.rb scripts in Enveomics platforms [42], and confirmed with the outputs from automated multi-locus species tree analysis (autoMLST) [43], using default settings with bootstrapping performed by IQ-Tree Ultrafast Bootstrap Analysis (1000 replicates). The recommended species cut-off was 95% for ANI and ~ 70% for AAI indices [44].

In silico chemotaxonomic analysis was conducted using MicroScope. Specifically, comparative analysis of MicroCyc metabolic pathways was performed using the genome of *C. ulmosensis* CV2^T^ against available PkGDB genomes of other *Cupriavidus* spp.

### Soil DNA extraction and qPCR

To evaluate the abundance of our isolates in the total microbial composition, amplicon sequencing was performed on the soil DNA from the sites where the strains were isolated. All DNA extractions were performed using three soil replicates. Total DNA from 2.25 g 2015 and 1917 soil samples (*n* = 3) was extracted using a DNeasy PowerSoil Pro kit (Qiagen) according to the manufacturer’s instructions. DNA extracted from soils was cleaned using a DNeasy PowerClean Pro Cleanup kit (Qiagen) and stored at −20 °C until ready for use.

Quantitative PCR (qPCR) primers were designed specific to *coxL* from *C. ulmosensis* CV2^T^ and *Pb. terrae* COX using Primer3 (version 3.0.1, https://Primer3.org) with follow-up testing using primerBLAST with the respective genome sequences included to ensure specificity and that no non-specific products < 1,000 bp were predicted. OligoAnalyzer (Integrated DNA Technologies) was used to ensure the lack of predicted hairpins ∆G < −2.0 kcal/mol and the lack of self-dimers ∆G <  − 6.0 kcal/mol. The only exception was COX-*qcoxL*_rev, which was predicted to self-dimerise more than 4 bp from the 3’ end (∆G >  − 6.3 kcal/mol) and thus was accepted. Heterodimer analysis thresholds were set at ∆G ≥ −6.0 kcal/mol within 4 bp of the 3’ end. Primer sequences were as follows: CV2-q*coxL*_fwd (5’- GGCTGCTCTCGTCTATCCCG-3’) and CV2-q*coxL*_rev (5’-TGCCACGATGGACACCACAT-3’); COX-q*coxL*_fwd (5’-CCAGTTCAAGTCGGTCAAGGA-3’) and COX-q*coxL*_rev (5’-CACCGAATGGGAACGTGAAG-3’). The reaction mixture was prepared using 10 µl SensiFast SYBR Hi-Rox (Meridian Bioscience, Nottingham, UK), 0.8 µl of each primer (10 µM), 5–15 ng soil DNA/1 µl of *coxL* DNA standard, and water to 20 µl. qPCR was run using a Step 1 Plus instrument (Applied Biosystems, software version 2.2.2). Cycle conditions were as follows: 95 °C for 10 min, 30 cycles (95 °C for 1 min, 65 °C for 30 s, 72 °C for 30 s, reading at 82 °C for 10 s), then melt curve analysis (95 °C for 15 s, 60 °C for 1 min, then stepwise increases of 0.3 °C to a final 95 °C with reading for 15 s). Melt curve analysis produced single peaks from known standards (below) at Tm 87.4 °C for COX-q*coxL*_fwd and COX-q*coxL*_rev, and Tm 88.4 °C for CV2-q*coxL*-fwd and CV2-q*coxL*_rev reactions. *coxL* gene abundance in environmental samples was calculated against standards, which contained known numbers of copies of *coxL* from *C. ulmosensis* CV2^T^ or *Pb. terrae* COX, generated by PCR using Q5 High-Fidelity DNA polymerase (New England Biolabs, Hitchin, UK). Primers were designed to amplify the whole *coxL* gene from each strain using genomic DNA as a template. Primer sequences were as follows: CV2-*coxL*_fwd (5’-TCATCAGCCTGTCCTCAGGTTC-3’) and CV2-*coxL*_rev (5’-CACTGATCCGCAATGATCCCTC-3’); COX-*coxL*_fwd (5’-ACACGTCATGGGCAATCTCG-3’) and COX-*coxL*_rev (5’-GTACAACCTTCATGCGTGTCTC-3’). qPCR reaction efficiencies were as follows: CV2-*coxL*: 102.59% (R^2^: 0.999); COX-*coxL*: 100.31% (R^2^: 0.999). *coxL* copy numbers in environmental samples were normalised per gram of soil included in the original DNA extractions.

#### Total microbial community

Soil DNA was extracted as described in Sect. “Soil DNA extraction and qPCR”. Approximately 2.5 µg of DNA from the 1917 soil layer and ~ 85 ng of DNA from 2015 tephra was sent for 16S rRNA gene sequencing to Novogene UK. DNA was amplified with primers 341F ﻿(5’-CCTAVGGGRBCCASCAG-3’) and 806R (5’-GGACTACNNGGGTATCTAAT-3’) and 16S rRNA gene sequencing was done using Illumina PE250 at Novogene, UK. For the 16S sequence analysis, LotuS2 version 2.19 [45] was used in short read mode, using default quality filtering on Galaxy Europe (https://usegalaxy.eu). Raw 16S rRNA gene amplicon reads were quality filtered to ensure a minimum length of 170 bp; no more than eight homonucleotides, no ambiguous bases, average quality ≥ 27; and an accumulated read error < 0.5 vis sdm [46]. Filtered reads were clustered using DADA2 into amplicon sequence variants (ASVs) [47]. Postprocessing included uchime de novo and reference-based chimera removal [48], as well as back mapping operational taxonomic unit (OTU) sequences to a phi-X database for off-target removal [49]. The taxonomy to ASVs was assigned by RDP classifier (confidence threshold 80%) [50]. Abundance matrices were normalized using the rarefaction toolkit (RTK) [51].

For alpha-diversity, Shannon index, Simpson, the number of different species observed, and Species evenness were carried out using vegan package (version 2.6–6.1) in R (version 4.3.3). Alpha-diversity indexes were calculated based on the lowest number of sequences available (i.e., 74,386, subsampled using the *rarefy* function in vegan).

#### *coxL* amplicon sequencing

Soil DNA was extracted as described in Sect. “Soil DNA extraction and qPCR”, then *coxL* amplicon sequencing was performed on pooled environmental DNA by MRDNA Molecular Research (Shallowater, TX, USA). Triplicate soil DNA extractions were pooled in equal volume to a final 30 µl volume (1917: ~ 2.5 µg; 2015: ~ 85 µg). Primers specific to form-I *coxL* [3], with a barcode on the forward primer and a linker primer sequence GGCGGCTTYGGSAASAAGGT, were used according to King [3]. Amplicon sequencing was performed using DNA extracted from the 2015 (Barcode: ACTCAACA) and 1917 (Barcode ACTGATGT) deposits. PCR products were purified using Ampure PB beads (Pacific Bioscienes) and SMRTbell libraries (Pacific Biosciences) and sequencing was performed on the PacBio Sequel according to the manufacturer’s instructions. Sequences were depleted of barcodes and primers and short sequences < 150 bp were removed. Sequences with ambiguous base calls were also removed. Operational taxonomic units (OTUs) were defined by clustering at 3% divergence (97% similarity) followed by removal of singleton sequences and chimeras using USEARCH [52]. To generate the specific *coxL* gene classifier, we used QIIME ‘qiime feature-classifier fit-classifier-naive-bayes command [53]. The classifier was trained using a curated custom database that included reference sequences and taxonomic files based on NCBI classification [54].

#### Statistical analysis

Statistically significant differences between conditions for CO consumption tests were compared in triplicate by one-way analysis of variance (ANOVA) followed by a Tukey post hoc test (included in R version 4.3.2). Statistical significance of qPCR (in triplicate) and diversity data (in triplicate) were determined by using Welch’s 2-sample T-test (package datarium version 0.1.0 (https://rdrr.io/cran/datarium/)).

## Results

### Targeted enrichment and isolation of carboxydovores

A modified enrichment strategy was used to isolate carboxydovores from volcanic strata formed after eruptions in 2015 (tephra) and 1917 (soil). Samples of the 1917 and 2015 strata consumed ~ 100 ppmv CO after 48 and 72 h, respectively, while initial slurries and sub-cultured enrichments continued consuming CO throughout 700-h incubations. After enrichment, two strains were successfully isolated, *Cupriavidus ulmosensis* CV2^T^ and *Pb. terrae* COX, isolated from 1917 and 2015 volcanic stratifications, respectively. *C. ulmosensis* CV2^T^ is a Gram-negative, rod-shaped aerobic heterotrophic bacterium. *Pb. terrae* COX is also a Gram-negative, rod-shape aerobic heterotrophic bacterium.

**Fig. 1:**
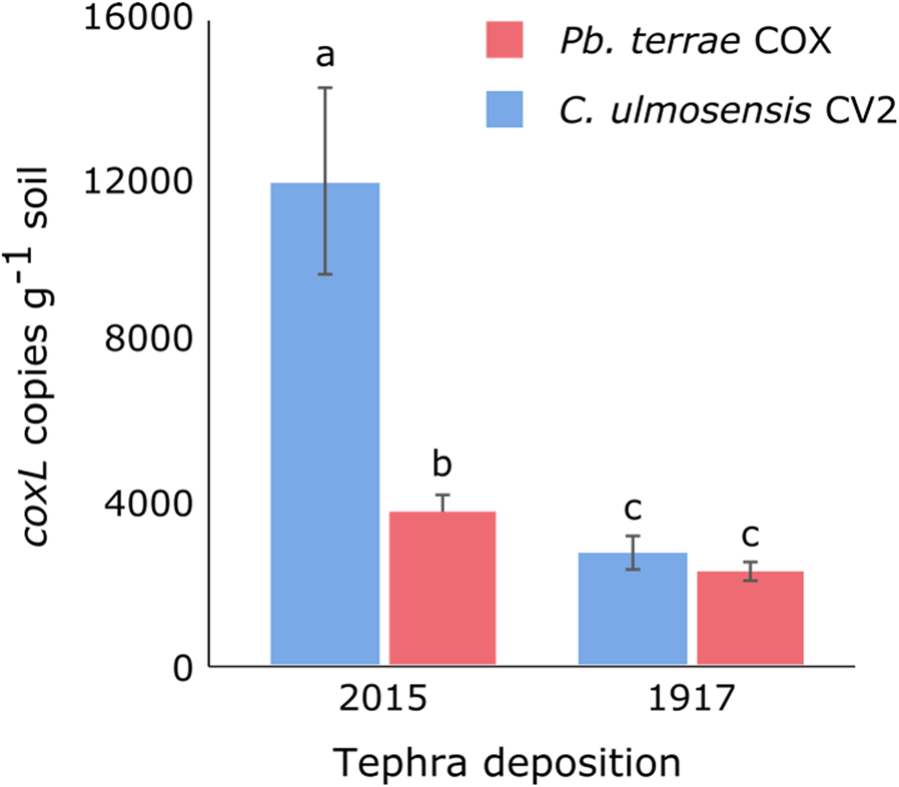
Abundance of *coxL* genes specific to *C. ulmosensis* CV2^T^ and *Pb. terrae* COX in 2015 and 1917 volcanic deposits, determined by qPCR, normalised per gram of the soil/tephra material. Bars represent mean values with standard deviation of independent triplicates for each tephra layer. Differing letters denote statistically significant differences between the samples (*p* ≤ 0.05), determined by Welch’s T-test

qPCR specific to *coxL* from *C. ulmosensis* CV2^T^ (CV2-*coxL*) and *Pb. terrae* COX (COX-*coxL*) was used to determine the abundance of these strains in the deposits from which they were isolated. The *coxL* gene associated with both *C. ulmosensis* CV2^T^ and *Pb. terrae* COX was significantly less abundant in the 1917 soil layer than the 2015 tephra layer (*p* ≤ 0.05) and were present at similar levels within the 1917 soil layer. In the 2015 tephra, 12,000 ± 2,300 copies CV2-*coxL* g^−1^ were present, which was significantly higher than the 3,800 ± 400 copies COX-*coxL* g^−1^ found in the 1917 soil layer (*p* ≤ 0.05) (Fig. 1).

### Genome sequence analysis of novel carboxydovores

Strains *C. ulmosensis* CV2^T^ and *Pb. terrae* COX are similar to *Cupriavidus basilensis* strain DSM 11853^ T^ (99.33% nucleotide ID with 16S rRNA, BioSample ID SAMN12697569, Bioproject PRJNA563568) and *Pb. terrae* strain KU-64^ T^ (NBRC 100964, 99.73%, BioSample ID SAMD00391168, Bioproject PRJNA33175), respectively. The genome of *C. ulmosensis* CV2^T^ is over 10 Mbp with a GC content of 64.8% (Table S3). The genome of *Pb. terrae* COX is also over 10 Mbp with a GC content of 62.0%. Additional genome information is listed in Table S3. *C. ulmosensis* CV2^T^, with an ANI of 92.3% and a dDDH of 47.5% to the closest type strain *C. basilensis* strain DSM 11853^ T^, was determined to be a member of a new species (Figures S2; S3), corroborated by autoMLST analysis. *Pb. terrae* COX had an ANI of 96.42% and a dDDH of 71.1% to the closest type strain *Pb. terrae* strain KU-64^ T^ (NBRC 100964), confirming it to be *Pb. terrae* (Figures S4; S5).

**Fig. 2:**
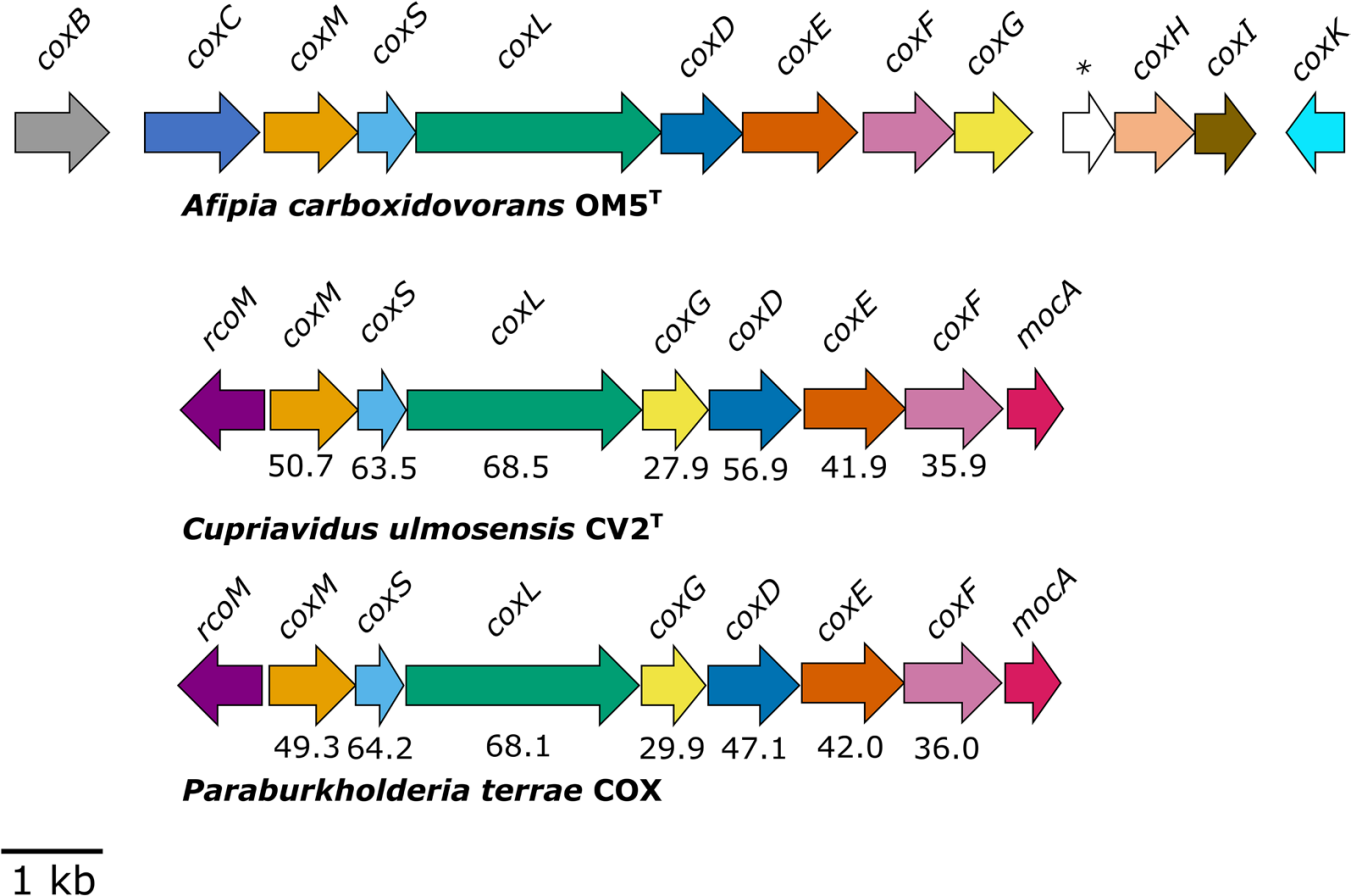
Form-I CODH-encoding gene clusters from *C. ulmosensis* CV2^T^ and *Pb. terrae* COX. Translated amino acid identities (%) were calculated using BLASTp against homologous query sequences from the model type strain carboxydotroph *Afipia carboxidovorans* OM5^T^. Asterisk (*) indicates a hypothetical gene

**Fig. 3:**
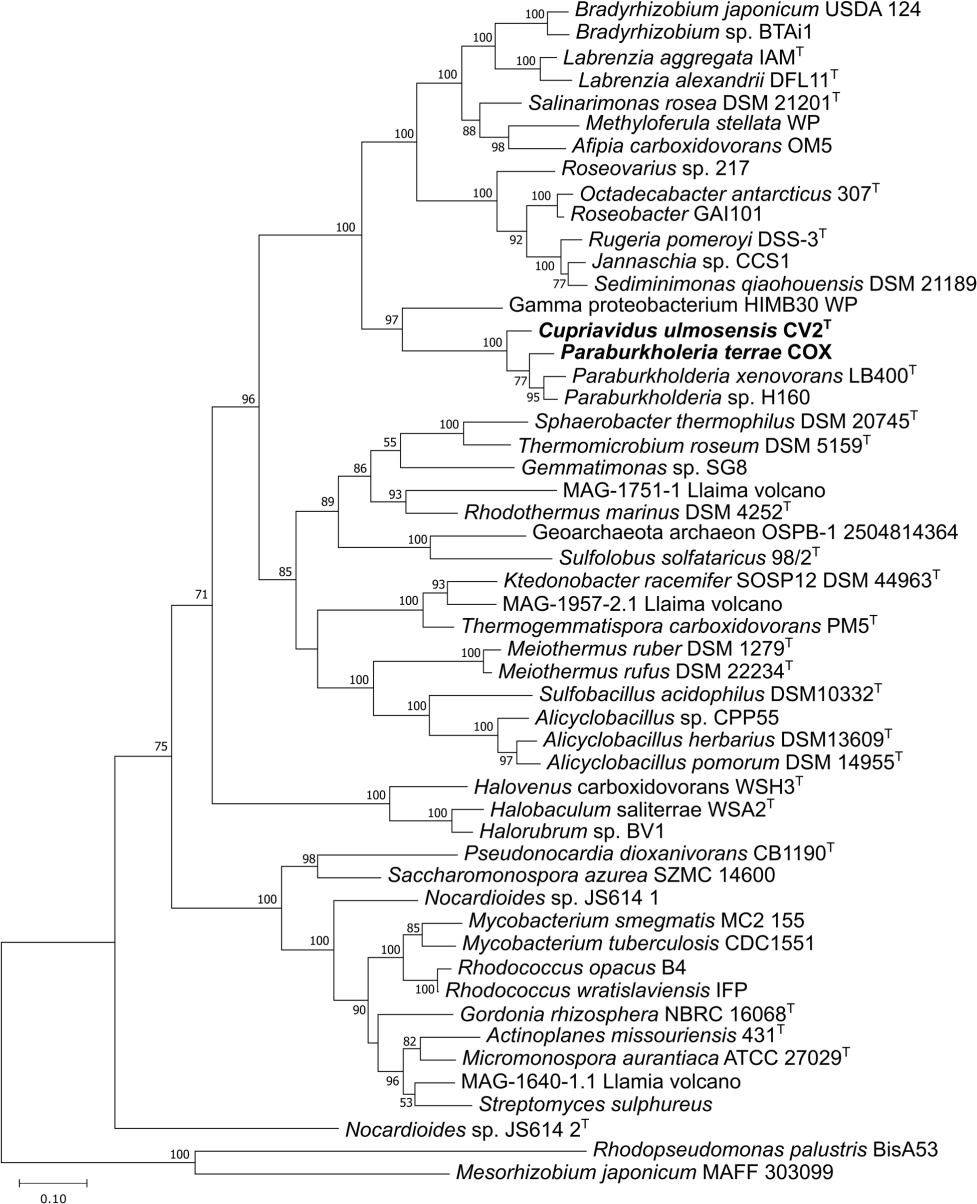
Evolutionary relatedness of translated CoxL amino acid sequences. The tree was drawn using the Maximum Likelihood method with 500 Bootstrap replicates in MEGA11 [39]. MAGs were retrieved from Hernández et al. [71]

The genomes of *C. ulmosensis* CV2^T^ and *Pb. terrae* COX contain a large number of coding sequences (10,380 and 10,390, respectively). Subsystems analysis indicated various differences in the functional capacity of each strain; *Pb. terrae* COX had far more genes encoding roles in motility and chemotaxis, carbohydrate metabolism, and sulfur metabolism, while *C. ulmosensis* CV2^T^ had more genes encoding roles in membrane transport, fatty acid metabolism and metabolism of aromatic compounds (Table S3). Individual clusters of three genes were identified on each genome that had homology with the form-I CODH (*coxMSL*) cluster of *A. carboxidovorans* OM5^T^ (Fig. 2), and form-II CODH-encoding genes were also identified (*coxSLM*) in both bacteria. Inferred CoxL amino acid sequences from *C. ulmosensis* CV2^T^ and *Pb. terrae* COX were most closely related to other Pseudomonadota CoxL sequences (Fig. 3). *C. ulmosensis* CV2^T^ and *Pb. terrae* COX only possess a limited selection of the accessory *cox* genes (*coxGDEF*), which were initially identified in the CODH-encoding gene cluster from *A. carboxidovorans* OM5^T^ (Fig. 2). Additionally, two distinct genes were present in the novel strains when compared to *A. carboxidovorans* OM5^T^; *rcoM*, encoding a putative transcription factor, and *mocA*, which encodes a molybdenum cofactor cytidylyltransferase (Fig. 2). The genome of *C. ulmosensis* CV2^T^ indicated a capacity for trace gas utilisation beyond CO alone. A gene cluster encoding a putative Ni–Fe hydrogenase was detected, which shared the same genetic organisation with the hydrogen-oxidizing bacterium *C. necator* H16 (Figure S6). *Pb. terrae* COX lacked any recognisable hydrogenase-encoding genes on its genome.

Genes with predicted roles in stress responses were analysed on the genomes of *C. ulmosensis* CV2^T^ and *Pb. terrae* COX using the MicroScope genome annotation platform. Both strains possessed *lexA* genes, involved in the transcriptional regulation of SOS responses. Desiccation resistance was suggested, as both strains possessed *otsAB* genes involved in trehalose biosynthesis. Resistance to, or stress responses against, elevated temperatures was not suggested by the genomes of either strain, as very few heat shock protein-encoding genes were detected or genes encoding known chaperones involved in temperature responses, such as GroEL.

The genome of *C. ulmosensis* CV2^T^ was further studied in comparison to previously reported *Cupriavidus* spp. to identify key areas of chemotaxonomic separation (Tables S4–S11). Amino acids biosynthesis reactions predicted by the genome analysis were similar among the compared species, as were carbohydrates biosynthesis, cell structures biosynthesis, fatty acids and lipids biosynthesis. *C. ulmosensis* CV2^T^ had a similar number of predicted reactions for the Calvin-Benson-Basham cycle as *C. necator* N-1^ T^, which possess a full operon for autotrophic CO_2_ assimilation.

### Physiological and metabolic characterisation

*C. ulmosensis* CV2^T^ and *Pb. terrae* COX grew using a relatively wide range of organic carbon sources (Table S2). However, *C. ulmosensis* CV2^T^ was unable to use most of the sugars tested in this study, showing only weak growth with arabinose. In contrast, *Pb. terrae* COX showed growth on a broader range of organic compounds (Table S2). Similar temperature profiles were exhibited by each strain (Table S2). *C. ulmosensis* CV2^T^ grew to the same final OD_600_ (0.51–0.53) at 25–30 °C, to a substantially lower OD_600_ at 37 °C (0.15), and failed to grow at 45 °C. *Pb. terrae* COX reached a slightly higher OD_600_ at 25 °C (0.40) than at 30 °C (0.34). Growth at 37 °C was slower (OD_600_ = 0.22) but consistent, while no growth was observed at 45 °C. *C. ulmosensis* CV2^T^ grew at 0–1% NaCl and failed to grow at 10% NaCl, while 1% NaCl appeared to stimulate growth of *Pb. terrae* COX (OD_600_ = 0.48), but 10% NaCl inhibited growth entirely (Table S2).

**Fig. 4:**
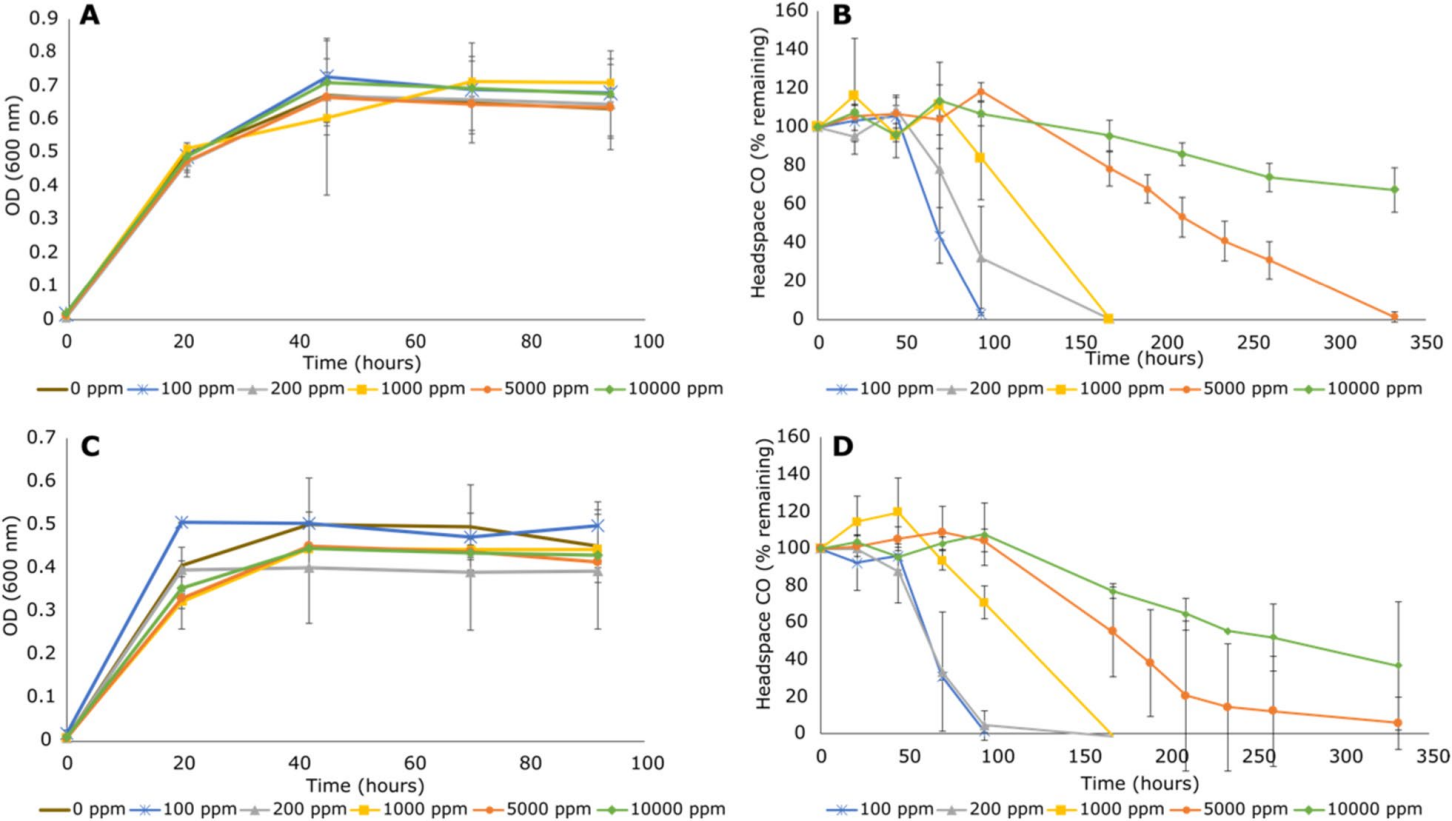
Growth of, and CO consumption by, bacterial strains. When determining statistically significant consumption of CO during CO tolerance experiments, one-way ANOVA was used to compare all timepoints within a given concentration.** A**) Growth of *C. ulmosensis* CV2^T^ with 5 mM pyruvate combined with varying concentrations of headspace CO (0–10,000 ppmv) (OD_600_). ** B**) Headspace CO (% remaining vs. timepoint 0) during growth with 5 mM pyruvate by *C. ulmosensis* CV2^T^. ** C**) Growth of *Pb. terrae* COX with 5 mM pyruvate combined with varying concentrations of headspace CO (0–10,000 ppmv) (OD_600_). ** D**) Headspace CO (% remaining vs. timepoint 0) during growth with 5 mM pyruvate by *Pb. terrae* COX. Bars represent mean values with standard deviation of independent triplicate incubations for each substrate

Carboxydovory was confirmed as incubation of each strain with elevated concentrations of CO, with or without organic carbon, failed to facilitate growth (Figure S7; Figs. 4A, C). *C. ulmosensis* CV2^T^ grew on 5 mM pyruvate between pH 5.0–8.0 (Figure S8), and *Pb. terrae* COX grew between pH 5.0–7.0 (Figure S9). After 24 h, *C. ulmosensis* CV2^T^ had grown to a statistically similar OD_600_ at pH 6.0, 7.0 and 8.0, with a small but significant decrease in growth at pH 5.0 when compared to the other three conditions (*p* ≤ 0.05) (Figure S8). No statistically significant difference was detected after 48 h of growth. Additionally, no growth was observed at pH 4.0 over the course of the experiment by either strain. *Pb. terrae* COX grew to a significantly higher final OD_600_ at pH 7.0 than at any other pH (*p* ≤ 0.01) and remained significantly higher over the course of the experiment (Figure S9). No statistically significant difference in growth was observed between pH 5.0 or 6.0 over the course of the experiment, and no growth was observed at pH 4.0 or 8.0. A major physiological difference observed between the two strains was the production of copious amounts of extracellular polymeric substance (EPS) by *C. ulmosensis* CV2^T^, which influenced colony morphology on plates due to increasingly mucoid appearance over time and was visible under scanning electron microscopy (Figure S10).

### Consumption of elevated CO concentrations by carboxydovores

Both *C. ulmosensis* CV2^T^ and *Pb. terrae* COX consumed CO when grown in liquid culture with 5 mM pyruvate (Fig. 4A–D). 100 ppmv CO was consumed below detection by *Pb. terrae* COX after 5 days (Fig. 4D), with an initial lag period of approximately 2 days before CO was consumed. Similarly, *C. ulmosensis* CV2^T^ exhibited a lag period of approximately 2 days before consuming CO from a 100 ppmv headspace (Fig. 4B). CO uptake was observed between 100–10,000 ppmv CO (Fig. 4), but not at 100,000 ppmv CO (data not shown), and notable differences in the lag period before CO consumption began were observed for each strain at different concentrations of CO (0–10,000 ppmv). *C. ulmosensis* CV2^T^ consumed a significant amount of CO from the headspace of vials with 100 ppmv CO after 70 h (ANOVA: p ≤ 0.01), 200 ppmv CO after 94 h (*p* ≤ 0.001), 168 h for 1,000 ppmv CO (*p* ≤ 0.001), and 210 h for 5,000 ppmv CO (*p* ≤ 0.001) and 10,000 ppmv CO (1% v/v) (*p* ≤ 0.001) (Fig. 4B). *Pb. terrae* COX consumed a significant quantity of headspace CO after 70 h with an initial concentration of 100–200 ppmv (ANOVA: *p* ≤ 0.001), 168 h for 1,000 ppmv (*p* ≤ 0.01), 190 h for 5,000 ppmv (*p* ≤ 0.001) and did not consume a significant quantity of CO from the headspace with 10,000 ppmv (1% v/v) CO during the test period (Fig. 4D). *C. ulmosensis* CV2^T^ consumed a significant quantity of CO at 5,000–10,000 ppmv CO with a shorter lag period than *Pb. terrae* COX, while the latter strain exhibited the shortest lag period before consuming a significant quantity of CO at 100–200 ppmv initial concentration (*p* ≤ 0.01), with similar consumption of CO at higher concentrations (Fig. 4D).

### Regulation of CO consumption by growth conditions and growth phase

**Fig. 5:**
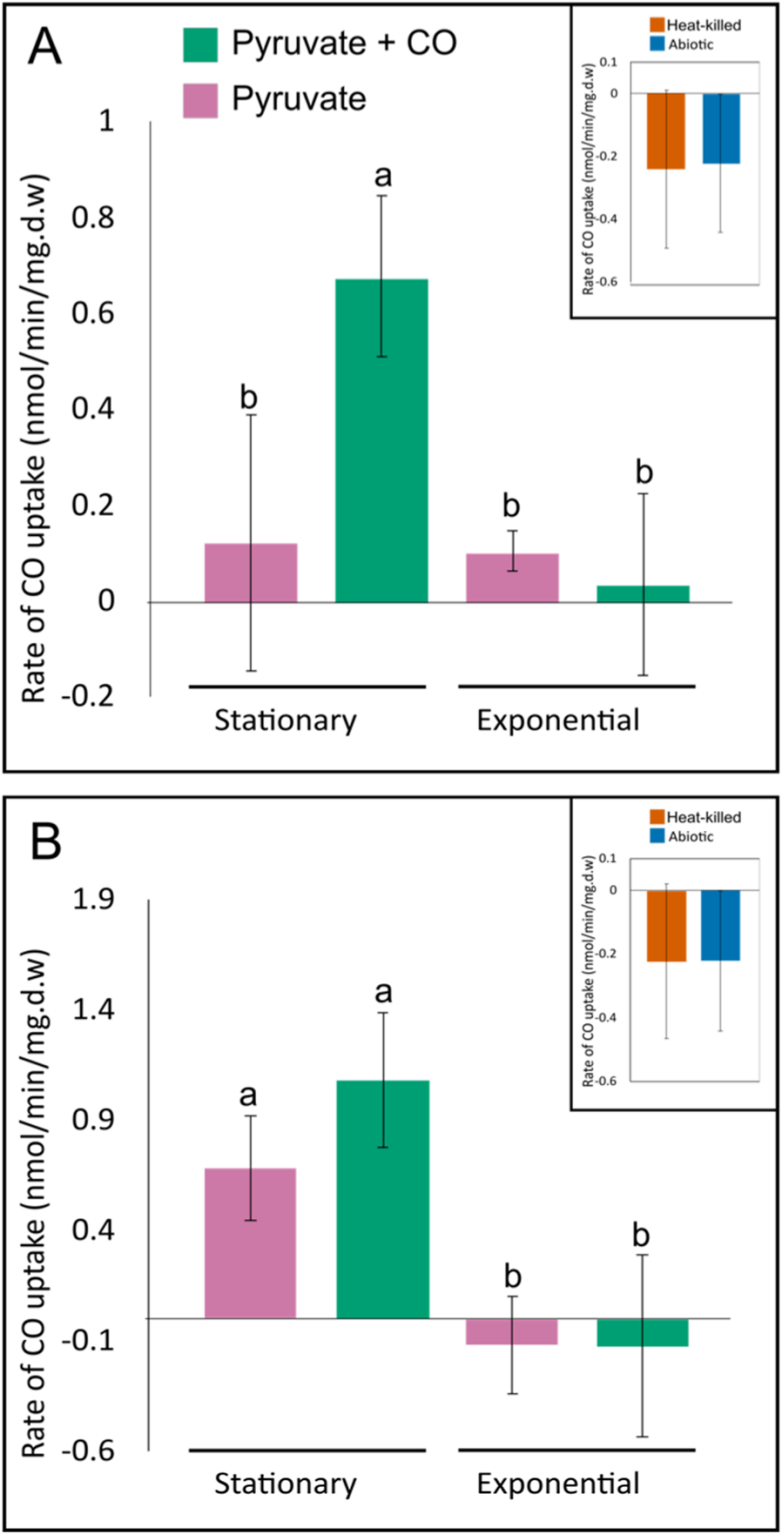
Rates of CO uptake (nmol/min/mg dry weight) by (**A**) *C. ulmosensis* CV2^T^ and (**B**) *Pb. terrae* COX at stationary phase or exponential phase, varied according to the growth substrate. Bars represent mean values with standard deviation of independent triplicate incubations for each substrate. Different letters above the bars indicate significant differences determined by one-way ANOVA followed by a Tukey post hoc test

The rate of CO uptake during exponential and stationary phase was compared (Fig. 5A, 5B). For both strains, the rate of CO oxidation increased significantly during stationary phase compared to exponential phase for cells, which were grown on a combination of pyruvate and CO (ANOVA: *p* ≤ 0.01). Little to no CO uptake was detected in cells harvested at exponential phase for either strain. *C. ulmosensis* CV2^T^ oxidised very little CO during stationary phase after growth with pyruvate alone (Fig. 5A), while *Pb. terrae* COX oxidised CO at a very similar rate during stationary phase regardless of the carbon sources available during growth (ANOVA: *p* ≥ 0.05) (Fig. 5B).

It was unknown how variations in pH might impact the ability of *C. ulmosensis* CV2^T^ and *Pb. terrae* COX to oxidise CO. *C. ulmosensis* CV2^T^ oxidised CO at 0.81 ± 0.10 nmol CO/min/mg.dw at pH 5.0, with a decrease in the rate of CO oxidation from 0.55 ± 0.12 to 0.36 ± 0.28 nmol CO/min/mg.dw at pH 6.0 and 7.0, respectively (Figure S11A). The rate of CO oxidation increased significantly to 1.22 ± 0.21 nmol CO/min/mg.dw at pH 8.0 (*p* ≤ 0.01 vs. pH 7.0). *Pb. terrae* COX exhibited similar rates of CO uptake at each tested pH, with rates of 1.57 ± 0.34 and 2.08 ± 0.55 nmol/min/mg.dw at pH 5.0 and 7.0, respectively, but the rate of CO uptake was significantly lower at pH 6.0, measuring 1.02 ± 0.29 nmol/min/mg.dw (*p* = 0.05 vs. pH 6.0) (Figure S11).

### Relative abundance of the Pseudomonadota and bacterial diversity in recent volcanic deposits

**Fig. 6:**
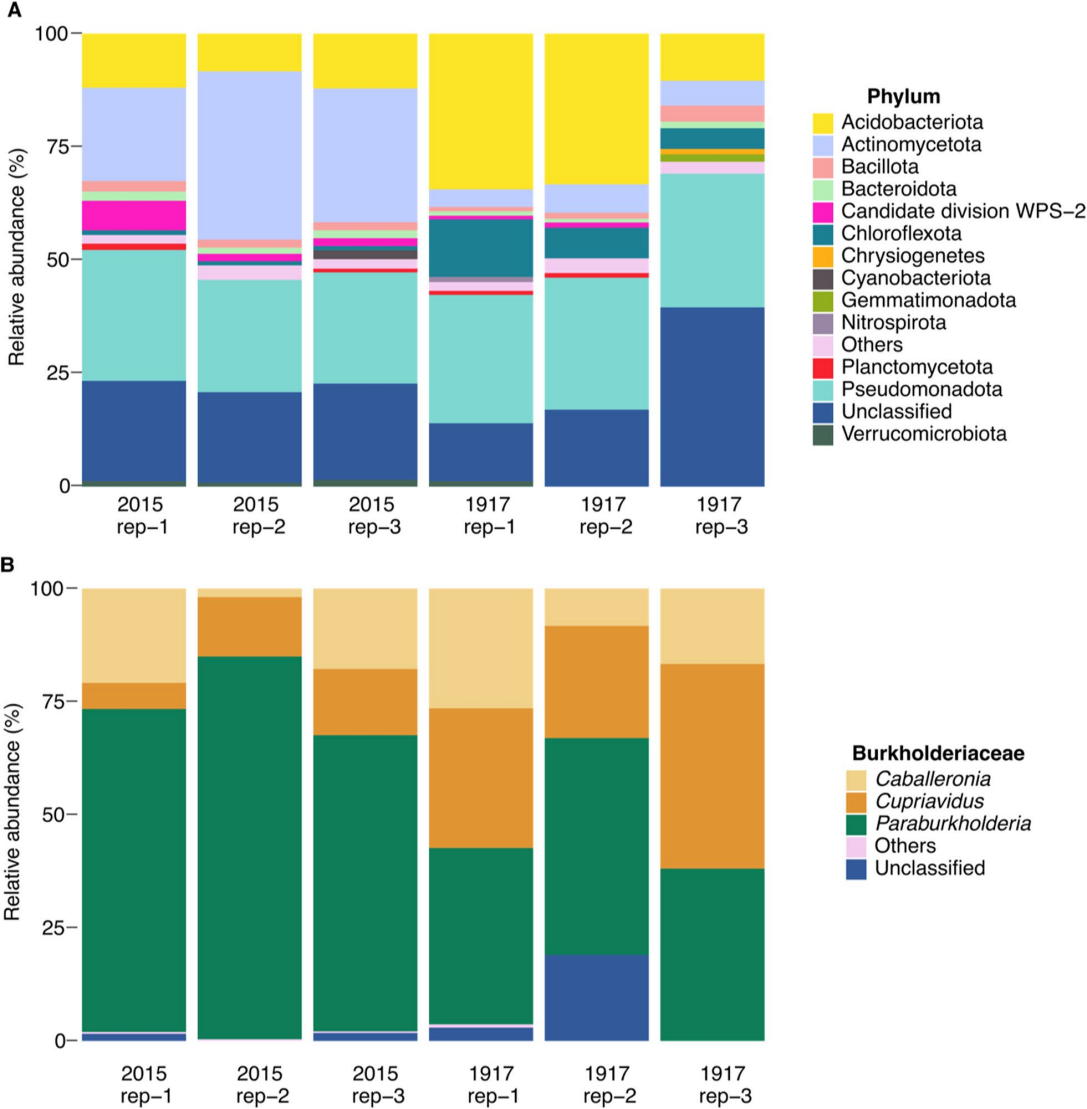
Relative abundance of 16S rRNA gene sequences in Calbuco stratifications originating from eruptions in 2015 and 1917. **A** Relative abundance of 16S rRNA of phyla. **B** Relative abundance of members of the Burkholderiaceae. Where the abundance of unclassified was lower than 0.8%, it was grouped into “Others”

Amplicon sequencing was conducted on soil DNA from the sites where the strains were isolated to assess the abundance of these two isolates within the overall total microbial community. The bacterial communities that inhabited the 2015 and 1917 volcanic deposits were composed largely of Pseudomonadota (26.08% and 28.99% average relative abundance (RA), respectively), Actinomycetota (29.09% and 5.21% RA, respectively) and Acidobacteriota (10.79% and 26.02% RA, respectively) (Fig. 6A). Other groups in the context of CO oxidation included the Chloroflexota, which comprised 0.96% of the community in the 2015 tephra layer, compared to 8.01% in the 1917 layer.

Given that *C. ulmosensis* CV2^T^ and *Pb. terrae* COX are members of the Pseudomonadota, this group was studied in greater detail. Alphaproteobacteria dominated both the 2015 and 1917 volcanic deposits (32.97% and 34.99% relative abundance (RA), respectively), followed by Betaproteobacteria (12.22% and 18.01% RA, respectively) (Figure S12). The Burkholderiaceae comprised 32.54% and 5.26% of the Betaproteobacteria in the 2015 and 1917 strata and were dominated by the genus *Paraburkholderia* in the 2015 volcanic deposit (73.81% average RA), with a lower RA in the 1917 deposit (41.67% RA) (Fig. 6B). *Cupriavidus* spp. accounted for less of the Betaproteobacteria in the 2015 volcanic deposit (11.17% RA) but were greater at 33.64% RA in the 1917 volcanic deposit (Fig. 6B). The Shannon and Simpson indices of 16S rRNA nucleotide diversity were very similar across soil samples from both tested strata (Table S12), with no significant differences observed between the two sites analysed (*p* > 0.05 for Shannon, Simpson and Evenness). Although these differences were not statistically significant, 1917 soil (replicate 1) had the highest richness, indicating that it hosted the most species, while 1917 soil (replicate 3) hosted the least. In contrast, 2015 tephra (replicate 1) had the highest Shannon diversity (5.53), suggesting a more diverse ecosystem, whereas 1917 soil (replicate 3) had the lowest (4.52). This aligns with the Simpson values, where 2015 tephra (replicate 1) had the highest value (0.99), indicating a more diverse community, and 1917 soil (replicate 3) had the lowest (0.95). Additionally, 2015 tephra (replicate 1) had the most even community (0.76), while 1917 soil (replicate 3) had the least (0.65).

Community-wide analysis was conducted to determine how the CO-oxidising bacterial population changed over time following eruptions. Sequencing of *coxL* amplicons was used as a proxy for investigating the CO oxidiser community; *coxL* OTUs relating to the phylum Pseudomonadota were the most abundant in the 1917 (28.48%) soil layer, while the majority of *coxL* OTUs derived from the 2015 sediment were related to the Actinomycetota (60.41%) (Figure S13A). *coxL* OTUs relating to the Betaproteobacteria were less abundant than the Alphaproteobacteria in the 2015 tephra layer (Figure S13B) where CV2-*coxL* abundance was highest (Fig. 1). Betaproteobacteria *coxL* OTUs were slightly more abundant in the 2015 tephra layer than the 1917 soil layer (Figure S13B), with Betaproteobacteria OTUs in the 2015 layer corresponding entirely to the order Burkholderiales (Figure S13C). Burkholderiales *coxL* OTU abundance was similarly dominant in the 1917 soil layer. Within this order, the Burkholderiaceae were more abundant in the 2015 tephra layer (Figure S13D), although the Comamonadaceae were more abundant in the two strata. Collectively, OTUs corresponding to the Burkholderiaceae accounted for 0.1% of all OTUs. Twenty-four *coxL* OTUs related to the Burkholderiaceae were further analysed using BLASTn, with the highest identity sequences (78.34–98.79% nucleotide identity) belonging to the genera *Burkholderia* and *Paraburkholderia*. When alignments of translated CoxL amino acid sequences were phylogenetically analysed using the Maximum Likelihood method, one OTU was found to be closely related to CoxL from *C. ulmosensis* CV2^T^ (OTU_6625) (Figure S14), while CoxL from *Pb. terrae* COX clustered most closely with OTU_12825.

## Discussion

In this study, soil samples were collected from a stratigraphic profile formed by several layers of volcanic eruptions at Calbuco volcano, Chile. The two isolates characterised in this study were isolated from the 2015 tephra and from the 1917 soil strata. The higher organic matter content in the 1917 soil was likely due to the historic use of this site for grazing cattle circa 1940–1998 [Barbara Corrales, Parque Volcánico Valle Los Ulmos, Chile, pers. Comm].

### CO-oxidising Pseudomonadota colonise volcanic tephra layers

In this study, conditions were adapted from previous enrichments [55] by using sufficiently low concentrations of pyruvate that CO consumption was unlikely to be inhibited [17, 56, 57], while providing 100 ppmv CO in regular doses as described previously [3] to reduce the likelihood of enriching carboxydotrophs, which are likely to use higher CO concentrations [2, 58]. Additionally, pH 5.5 (VL55 medium) was selected due to the use of this condition when enriching and isolating carboxydovores in previous work [5]. Rapid consumption of 100 ppmv CO was observed, confirming that the media composition was not inhibitory. This enrichment method led to the isolation of two members of the Pseudomonadota, *C. ulmosensis* CV2^T^ and *Pb. terrae* COX, a group that was previously noted for being highly abundant in vegetated volcanic deposits [17, 21, 59]. Pseudomonadota *coxL* gene abundance was positively correlated with increasing organic matter availability in vegetated volcanic soils [17], indicating the relevance of this phylum to environmental CO uptake.

*Pb. terrae* COX is a member of a well-known genus of CO-oxidising bacteria. CO-oxidising *Burkholderia* spp. and *Paraburkholderia* spp. have been isolated from many locations including >100-year-old vegetated deposits from Kilauea Volcano [55, 60]. To our knowledge, *Pb. terrae* has not been previously identified as a CO oxidiser. Conversely, *Cupriavidus* spp. is best known for autotrophy and, although a volcanic isolate is available [61], no CO oxidising species has previously been identified, even when form-II CODH was present [2].

### Colonisation of volcanic deposits informed by genome sequencing

The form of CoxL was determined by identifying the characteristic form-I active site motif AYXCSFR [21] during alignment of the translated amino acid sequences with known form-I CoxL sequences (Fig. 3). Many BLAST results for CV2-*coxL* corresponded to form-II *coxL* in other *Cupriavidus* spp. (Table S13), indicating that CO oxidation using form-I CODH may be unusual in the genus *Cupriavidus*. *Cupriavidus* sp. UME74 (Genbank NUA31334.1), isolated from Portuguese forest soil, and *Cupriavidus* sp. SK-3, isolated from lagoon sludge contaminated by polychlorinated biphenyls [62], contain form-I *coxL* but no evidence of CO oxidation has been reported. The genetic layout of the *cox* gene clusters in *C. ulmosensis* CV2^T^ and *Pb. terrae* COX were identical, sharing a limited number of accessory *cox* genes (*coxGDEF*), *rcoM*, encoding a putative heme-containing CO-sensing transcription factor [63, 64] and *mocA*, encoding a molybdenum cofactor cytidylyltransferase that may facilitate the assembly of the molybdopterin cytosine dinucleotide (MCD) cofactor required by CODH [65] (Fig. 2).

Carboxydovory was indicated by the genomes of *C. ulmosensis* CV2^T^ and *Pb. terrae* COX due to the lack of a complete CO_2_ fixation pathway. However, each genome encodes a phosphoenolpyruvate carboxylase, meaning that assimilation of a small amount of carbon from CO through anaplerotic CO_2_ fixation following CO oxidation cannot be discounted [66]. Additional capacity for trace gas oxidation was indicated by the genome of *C. ulmosensis* CV2^T^ due to the presence of a hydrogenase gene cluster. The ability to gain energy from the oxidation of both CO and H_2_ may provide a competitive advantage to carboxydovores over heterotrophs or other mixotrophs during the colonisation of volcanic deposits [67]. Hydrogenases are widely distributed in nature and can support microbial productivity when organic carbon is limited [68, 69], and their activity has been observed in volcanic cinders [70]. Evidence from genomes and studies with cultured isolates have demonstrated the use of both CO and H_2_ by many carboxydotrophs and carboxydovores [2, 13, 58, 67, 71, 72], suggesting that the use of both trace gases may be a relatively common strategy. *Pb. terrae* COX lacked a hydrogenase gene cluster, possibly contributing to the lower COX-*coxL* gene abundance observed compared to CV2-*coxL* in the 2015 tephra layer (Fig. 1) where organic carbon was lacking.

Biosynthetic and catabolic reactions predicted by the genome of *C. ulmosensis* CV2^T^ were similar to those of other *Cupriavidus* spp. during MicroCyc analysis (Tables S4-S11). All six *Cupriavidus* spp. lacked many possible carbohydrate degradation genes, including glucose catabolism (Table S10), consistent with the inability of *C. ulmosensis* CV2^T^ and other *Cupriavidus* spp. to grow using sugars (Table S2) [73]. An exception is *C. basilensis* OR16, which possesses a glucose dehydrogenase gene, permitting growth on glucose [74]. *C. ulmosensis* CV2^T^ possessed many genes with predicted roles in hydrogen oxidation, which were not present in the compared *Cupriavidus* spp. genomes (Table S11; Figure S6). H_2_-oxidation is not universal in *Cupriavidus* spp., even closely-related ones, as the autotrophic *C. necator* H16 grows using H_2_ and CO_2_ [75] while *C. necator* N-1^ T^, which possesses a full operon for autotrophic use of CO_2_, possesses no genes for hydrogen oxidation, potentially preventing growth using trace gases alone [76]. Perhaps the most compelling chemotaxonomic marker to delineate *C. ulmosensis* CV2^T^ from other *Cupriavidus* spp. is the ability to oxidise CO to CO_2_, the presence of form-I CODH, given the apparent rarity of form-I *coxL* across other isolated *Cupriavidus* spp. genomes, and the lack of autotrophic growth. Form-I CODH has not been mechanistically linked to CO oxidation in *C. ulmosensis* CV2^T^. Studies disagree on the role of form-II CODH (characterised by active site motif AYXGAGR), with some posing the form-II enzyme as an active CODH [77] and others suggesting inactivity [78]. Both *Pb. terrae* COX and *C. ulmosensis* CV2^T^ possess the form-II CODH gene cluster (*coxSLM*), but the most likely source of CO oxidation is the form-I enzyme in each case, as a previous study of CO oxidation in strains possessing both form-I and form-II CODH-encoding gene clusters vs. strains possessing only form-II concluded that those possessing form-II alone could not oxidise CO [78].

### Carbon utilisation, temperature, salt and pH tolerance of volcanic carboxydovores

Although the genus *Cupriavidus* can use a wide range of growth substrates, including sugars in some cases [79–81], *C. ulmosensis* CV2^T^ was unable to grow using any of the sugars tested except arabinose, likely due to the lack of a gene encoding phosphofructokinase, as determined by KEGG analysis on the MicroScope annotation platform. Similarly, *C. basilensis* strain DSM 11853^ T^ was unable to use glucose [73] as were nine of the 10 volcanic *Cupriavidus* spp. strains isolated from mud flows [61]. *Pb. terrae* COX grew well with most organic carbon sources tested, likely due to an intact glycolysis pathway permitting sugar utilisation, indicating that this strain would be the most effective at scavenging organic carbon sources in the environment. However, this apparent metabolic advantage was not reflected by the abundance of *Pb. terrae* COX when compared to *C. ulmosensis* CV2^T^ (Fig. 1). Of the two strains isolated in this study, *C. ulmosensis* CV2^T^ may be more likely to grow in changeable environments due to its broader pH tolerance (Figures S8; S9), but *Pb. terrae* COX may be more effective at employing a mixotrophic lifestyle under similar conditions due to the limited impact of pH on CO uptake (Figure S11). It is currently unclear whether changes in the rate of CO oxidation are due to the efficiency or expression of CODH at each pH. The lack of growth at acidic pH 4.0 was surprising, given that volcanic ash can significantly contribute to soil acidification [20]. However, the pH levels of the environmental samples, 8.24 (tephra 2015) and 7.1 (soil 1917), may have contributed to the lack of adaptation to growth at lower pH. *Pb. terrae* COX was unable to grow at pH 8.0 (Figure S9), likely inhibiting the growth of this strain in the 2015 tephra layer due to the alkaline pH (Table S1), while *C. ulmosensis* CV2^T^ would be more likely to grow successfully under both pH conditions (Figure S8), potentially contributing to the higher abundance of this strain compared to *Pb. terrae* COX (Fig. 1).

Volcanic ecosystems present numerous challenges to resident microorganisms resulting from fluctuating temperature, desiccation, pH, among others. Both *C. ulmosensis* CV2^T^ and *Pb. terrae* COX grew similarly well at 25 °C and 30 °C but exhibited poor growth at 37 °C and no growth at 45 °C (Table S2). *C. basilensis* HMF14 was cultured up to 41 °C [82], suggesting a similar growth profile to *C. ulmosensis* CV2^T^*. Pb. terrae* strains isolated from temperate soils similarly had a temperature optimum of 28 °C [83], falling within the expected optimum temperature range of *Pb. terrae* COX (Table S2), and growth of these strains was inhibited above 2% NaCl. Similarly, growth was unimpeded between 0–1% (w/v) NaCl but was inhibited at 10% (w/v) NaCl (Table S2). *Cupriavidus* spp. have been reported to grow over a range of salinities, with some inhibited by as low as 0.5% NaCl while others tolerate 1.5% [84]. Collectively, these data suggest that both strains would be capable of growth under moderate conditions in the volcanic deposits, but that geothermal activity causing harsh temperature fluctuations, particularly in the upper stratum, would inhibit growth. The genomes of *C. ulmosensis* CV2^T^ and *Pb. terrae* COX suggested resistance to desiccation resulting from trehalose biosynthesis (encoded by *otsAB*), which was previously reported during the desiccation-induced responses of other soil bacteria [85]. *C. ulmosensis* CV2^T^ produced copious amounts of EPS during growth, also observed in *C. baslensis* HMF14 [82], which may contribute to the formation of biofilms that promote resistance to stressors such as desiccation and temperature, and sequesters cations from the soil [86]. Such adaptations may contribute to the significantly higher abundance of *C. ulmosensis* CV2^T^ in the exposed 2015 tephra layer (Fig. 1). Desiccation resistance would be particularly beneficial in the 2015 tephra layer due to the lack of soil matrix contributing to more stable water availability [87]. The genome of *Pb. terrae* COX possesses a *pgaABCD* gene cluster, predicted to form poly-beta-1,6-*N*-acetyl-D-glucosamine (PGA), a key component in the formation of some biofilms [88]. Furthermore, two copies of the operon *pelABCDEFG* were located on the genome of *Pb. terrae* COX with predicted roles in biofilm formation. *Pb. terrae* BS001 also possessed the *pga* and duplicated *pel* operons [88], suggested to improve the suitability of this strain for colonising soils.

### Oxidation of elevated concentrations of CO

The consumption of varying concentrations of CO by both strains further confirmed the use of carboxydovory. It was previously reported that carboxydovores were unable to grow using CO as the sole source of carbon and energy due to inhibition of CO oxidation at high concentrations of CO [57, 89], but the lack of any detriment to growth (Figs. 4A–C) at any tested CO concentration (Fig. 4B, D) in our isolates indicated that CODH activity may be inhibited by excessive CO concentrations, rather than any toxicity to broader cellular processes. The shorter lag period before *C. ulmosensis* CV2^T^ consumed a significant quantity of CO at 5,000–10,000 ppmv CO indicated that higher concentrations of CO are less inhibitory to CODH activity in this strain compared to *Pb. terrae* COX (Figs. 4B; 4D). *C. ulmosensis* CV2^T^ and *Pb. terrae* COX use higher concentrations of CO less effectively when compared to many carboxydotrophs [58, 90] and carboxydovores [91], and more effectively than marine CO oxidisers such as *Stappia aggregata* [57]*.* CO tolerance was previously demonstrated using the autotrophic non-CO oxidiser *C. necator* H16, which acquired tolerance to 50% (v/v) CO during laboratory evolutions, compared to the wild-type strain was inhibited by ≥ 15% (v/v) CO [92]. As CO inhibited autotrophic growth in *C. necator* H16 rather than CODH activity, it is unknown whether tolerance to elevated CO could occur in a similar laboratory evolution study with *C. ulmosensis* CV2^T^. Little information is available in the literature to indicate the typical level of tolerance to CO in *Paraburkholderia* spp. The capacity of *C. ulmosensis* CV2 and *Pb. terrae* COX for oxidation of elevated CO is higher than appears to be necessary as environmental CO concentrations typically range from 60–300 ppbv [3].

### Conservative and generalist regulation of CODH activity

Inhibition of CO uptake during growth on pyruvate (Figs. 4, 5) was consistent with previous reports of carboxydovores switching to trace gas oxidation during stationary phase [13], indicative of a starvation response. The differing regulation of CO oxidation by each strain, in spite of the apparent conservation in the *cox* gene clusters (Fig. 2), merits further study to better understand the mechanisms and regulation of carboxydovory in the environment. *C. ulmosensis* CV2^T^ oxidised little to no CO at stationary phase when grown using pyruvate alone, likely indicating that CODH expression by this strain requires the presence of CO at greater than atmospheric concentrations (60–300 ppbv, [3]). *Pb. terrae* COX, meanwhile, oxidised CO at stationary phase regardless of CO concentrations during growth, indicating that CO uptake by *Pb. terrae* COX may not be regulated by the presence of elevated CO but, rather, by the presence or absence of other organic carbon sources.

The regulation of CODH expression is highly variable between different carboxydotrophs and carboxydovores. CODH expression is constitutive in some carboxydotrophs such as *Pseudomonas carboxydoflava* and *Hydrogenophaga pseudoflava* [15, 93, 94], although *P. carboxydoflava* begins to use gases such as CO and H_2_ only as supplementary energy sources (mixotrophy) when supplied with heterotrophic substrates. This was suggested to support *P. carboxydoflava* in assimilating surplus organic carbon [15]. This differs from the proposed usage of CO as a supplementary energy source by carboxydovores such as *C. ulmosensis* CV2^T^ and *Pb. terrae* COX, as carboxydovory may support these strains when starved of organic carbon as suggested previously [1, 13]. The novel Calbuco strains behave more similarly to *S. aggregata* (carboxydovore) and *A. carboxidovorans* (carboxydotroph), as CO oxidation by these strains was reduced or eliminated by organic carbon [38, 57]. Weber and King [57] suggested that glucose (or glucose metabolites) exerted allosteric repression on CODH expression in *S. aggregata,* even though this bacterium could not grow on glucose. Overall, the repression or modulation of CO oxidation by organic catabolites is a common phenomenon in both carboxydotrophs and carboxydovores. Carbon catabolite repression appears to be the most likely explanation for the observed regulatory profiles observed in *C. ulmosensis* CV2^T^ and *Pb. terrae* COX (Fig. 5). Organic matter is moderately abundant in the 1917 soil layer (Table S1), but the bioavailability of this carbon is unknown and therefore cannot be certain to inhibit CO oxidation in situ. Furthermore, while organic matter is very low in the 2015 tephra layer (Table S1), transient inputs of organic matter through wet and dry depositions [67] could promote heterotrophic growth while inhibiting CO oxidation.

The energetic advantages of the CODH expression profiles (Fig. 5) are not yet fully understood. *C. ulmosensis* CV2^T^ may conserve energy by repressing CODH expression when ambient CO is absent, while *Pb. terrae* COX would benefit from transient increases in CO concentration immediately, particularly if atmospheric concentrations of CO can be consumed similar to other carboxydovores [1, 3]. In contrast, *C. ulmosensis* CV2^T^ would need to synthesise the CODH enzyme each time it is exposed to CO. In an ecological context, while *Pb. terrae* COX may be able to acquire supplemental energy through CO oxidation more readily during periods of starvation, the conservative regulation of CODH by *C. ulmosensis* CV2^T^ may make the latter strain better equipped to survive under certain conditions due to the smaller metabolic burden caused by expressing CODH less often (Fig. 5). As *C. ulmosensis* CV2^T^ was more abundant in the 2015 tephra layer (Fig. 1), this strain may be more suited to colonising the harsh volcanic ecosystem. Detailed analysis of the metabolism and physiology of other carboxydovores may provide greater insight into the mechanisms of control of CO oxidation, particularly in extreme and changing ecosystems such as volcanic deposits.

### Total microbial community

Further analyses were included to evaluate the relative abundance of these isolates within the total microbial composition at these sites. The abundance of the Pseudomonadota in the unvegetated 2015 tephra (Fig. 6A) was noteworthy as this group was also abundant in deposits from older/revegetated Llaima and Kilauea volcanoes [17, 59], consistent with the suggested correlation of this group with organic matter availability [17]. However, the Pseudomonadota were also the most abundant group in recent deposits from the Krafla volcanic field (Iceland) [95] and in deposits from Miyake-Jima (Japan) [96]. The similar diversity and richness indices of the 16S rRNA gene were intriguing when examined in the context of the increasing organic matter content between the 2015 and 1917 layers (Tables S1; S12). Weber & King [17] found that, as levels of organic carbon increased along a gradient of unvegetated to vegetated volcanic deposits, the microbial community was dominated by the Pseudomonadota in vegetated areas and the abundance and diversity of CO-oxidising bacteria was similarly greater. Conversely, deposits of varying age from Calbuco Volcano showed similar diversity indices (Table S12) and similar abundance of the Pseudomonadota despite increasing organic matter content (Fig. 6A; Table S1), coupled with lower abundance of the Actinomycetota and a greater abundance of the Myxococcota in the older deposit. Even within the Pseudomonadota, the alpha-, beta-, gamma- and delta- subgroups were similarly abundant between layers (Figure S12). A potential confounding explanation for the low observed diversity between layers is the possibility that members of the microbial community in the upper tephra layers could become vertically distributed through the lower strata, particularly when considering environmental factors such as rain.

The similar 16S rRNA gene diversity indices (Table S12) indicate that, although the community changes across the different strata (Fig. 6A), environmental factors were likely insufficient to drive substantial changes in microbial community diversity. The abundance of the Chloroflexota in the 1917 soil was also noteworthy, as this group was abundant in recent volcanic deposits at other sites [17, 59, 97] and both metabolic and metagenomic studies indicated the potential of this group for CO oxidation [5, 13, 71]. Previous studies have posited that the oxidation of trace gases such as CO is a key driver in colonising such ecosystems [59, 67, 98], an important step in soil succession.

16S rRNA gene sequencing demonstrated that the genus *Cupriavidus* was more abundant in the 1917 soil layer than the bare 2015 tephra (Fig. 6), which contrasts with strain-level data showing that *coxL* copies of *C. ulmosensis* CV2^T^ were more abundant in the 2015 layer (Fig. 1). Conversely, the genus *Paraburkholderia* was more abundant overall in the 2015 tephra layer (Fig. 6) but *coxL* copy numbers of *Pb. terrae* COX were significantly less abundant in this deposit and slightly less abundant in the 1917 soil (Fig. 1). Confounding the interpretation of *coxL* gene abundance through qPCR is the fact that multiple *coxL* gene copies could bias the observed abundance. Single copies of form-I *coxL* were present on the genomes of *C. ulmosensis* CV2^T^ and *Pb. terrae* COX. However, other strains, such as *A. carboxidovorans* OM5^T^, possess *coxMSL* on a plasmid [11], which could influence similar attempts at CO-oxidiser enumeration if multiple copies are present. Similarly, methods such as qPCR do not distinguish between DNA from living or dead cells, meaning that the true abundance of *C. ulmosensis* CV2^T^ and *Pb. terrae* COX could be lower than indicated (Fig. 1). A possible explanation for the lower abundance of CV2-*coxL* and COX-*coxL* g^−1^ soil in the 1917 soil layer compared to the 2015 tephra layer is that the increased availability of organic carbon would promote a more complex microbial community, but this is unlikely when considering the community diversity data (Table S12). Hernández et al. [59] found that total bacterial abundance was similar in Llaima Volcano deposits from 1957 (early soil formation, some lichen present), 1751 and 1640 (> 10–20 cm soil depth, plant colonisation) [99], but that microbial diversity was higher in the older deposits. King [67] found that increases in organic matter in sites with < 1% organic carbon led to a greater biomass of CO oxidising bacteria, lending a potential advantage over other heterotrophs.

*coxL* OTUs relating to the Pseudomonadota were similarly abundant between the 2015 and 1917 volcanic deposits (22.51–28.48%, respectively) (Figure S13A). By comparison, *coxL* genes relating to the Pseudomonadota comprised 2.6% of a *coxL* gene clone library from unvegetated deposits from Kilauea Volcano (Hawaii), compared to 70–75% for transitional and vegetated sites [17]. *coxL* from the Betaproteobacteria comprised 1.63 and 1.07% of Pseudomonadota-specific OTUs in the 2015 and 1917 deposits, respectively (Figure S13B). Weber & King [17] found that Betaproteobacteria *coxL* gene abundance was correlated with increasing vegetation in Kilauea Volcano deposits. Therefore, the consistently low abundance of Betaproteobacteria *coxL* OTUs in the Calbuco Volcano strata may be due to the lack of vegetation across the deposits. Many translated Calbuco *coxL* OTUs were grouped with known CoxL from *Burkholderia* and *Paraburkholderia* species (Figure S14), and the Burkholderiaceae were most abundant in the 2015 tephra (Figure S13D), contrary to previous observations of abundance increasing with vegetation [60]. The genus *Cupriavidus* spp. was far more abundant in the 1917 soil, although these data are unlikely to correspond to CO-degrading *Cupriavidus* spp. abundance as form-I *coxL* appears to be rare in this genus and only one *coxL* OTU closely related to *C. ulmosensis* CV2^T^ *coxL* was detected in this study (Figure S14). 25% of culturable bacteria from bare and sparsely vegetated volcanic deposits from Mt. Pinatubo (the Philippines) were *Cupriavidus* spp., demonstrating the ability of this genus to colonise such ecosystems, but isolates from this environment were autotrophs characterised for their ability to oxidise hydrogen rather than CO-oxidisers [61, 100, 101].

The disparity between 16S rRNA abundance (Fig. 6) and *coxL* abundance (Fig. 1; Figure S13) strongly indicates that 16S rRNA abundance cannot provide information about the local CO oxidising community. Supporting this is the fact that CO oxidising *C. ulmosensis* CV2^T^ was significantly more abundant in the bare 2015 tephra layer than the 1917 soil layer (Fig. 1), contrary to the total microbial community data (Fig. 6B). More work is required to understand the relationship between soil succession and the abundance of carboxydovores from the Pseudomonadota, as well as other groups. For example, Weber & King [17] demonstrated that *coxL* OTUs related to the Proteobacteria dominated vegetated sites, while *coxL* OTUs from this group are found in similarly moderate abundance in both the 2015 and 1917 Calbuco deposits (Figure S13A), potentially because of the similarly low levels of vegetation at the chosen sampling depth (Figure S1). Moreover, our classifier was unable to accurately identify members of the Burkholderiaceae to genus level when using *coxL* alone, meaning that the abundance of carboxydovores from the genera *Cupriavidus* and *Paraburkholderia* remains unclear. The curated custom *coxL* database used to construct the classifier contained only 56 sequences, which likely does not capture the full taxonomic diversity of *coxL* genes present in environmental samples. This limitation may contribute to reduced classification accuracy, as observed with members of the Burkholderiaceae, whose sequences may diverge significantly from those included in the reference database. No *coxL* OTUs from the Burkholderiaceae were identified as belonging to *Cupriavidus* spp. when analysed using BLASTn, but Maximum Likelihood analysis demonstrated that OTU_6625 (*Burkholderia anthina* 1CH1 according to BLASTn) was most closely related to CoxL from *C. ulmosensis* CV2^T^ (Figure S14). CoxL from *Pb. terrae* COX was most closely related to OTU_12825 (*Pb. terrae* KU-46 according to BLASTn), indicating that the well-known CO oxidising genus *Paraburkholderia* can be more confidently identified using *coxL* sequences alone. This is unsurprising given that many CO oxidising species of *Burkholderia* and *Paraburkholderia* have been identified, their genomes sequenced, and *coxL* OTUs identified in the environment [102].

CO flux may be influenced by the Actinomycetota, as this phylum was abundant relative to the entire bacterial community (Fig. 6A) and 60.4% of *coxL* sequences were associated to this phylum in the 2015 tephra layer (Figure S13). Similarly, *coxL* relating to the Actinomycetota comprised 31% of *coxL* genes in a deciduous forest soil [102]. Carboxydotrophs and carboxydovores have been isolated from this group [2], and *Mycobacterium* spp. have been the subject of physiological and genetics studies [1, 103], which demonstrated their ability to consume environmentally relevant concentrations of CO. However, Lalonde & Constant [102] found that 14 *coxL* OTUs related to *Mycobacterium* spp. were not significantly linked to the rate of CO oxidation in forest soils, suggesting that this group may not always play an important role, but *coxL* OTUs from the Actinomycetota were well correlated with CO oxidation rates [102]. This study further indicates the potential relevance of the Actinomycetota for environmental CO uptake. It is noteworthy to mention that storing soils at 4 °C for three months could present some challenges for molecular DNA analysis, as DNA can degrade over time. While 4 °C slows down degradation compared to room temperature, it may not fully prevent it for extended periods, potentially resulting in lower-quality DNA that could impact downstream analyses.

## Conclusions

This study demonstrates an effective strategy for enriching and isolating carboxydovore bacteria from volcanic soils. Two new strains are presented: *C. ulmosensis* CV2^T^, the first confirmed CO-oxidising member of this genus, and *Pb. terrae* COX, a member of a well-known group of CO-oxidising bacteria. Both new CO-oxidising isolates display metabolic flexibility like other carboxydovores, and the genomes of these strains indicate adaptations for surviving in harsh volcanic ecosystems. Both strains are capable of oxidising relatively high concentrations of CO (1% v/v), with consumption of CO observed down to relatively low levels specifically during stationary phase, suggesting that CO oxidation is used to gain supplemental energy during starvation. The regulations of CO oxidation by *C. ulmosensis* CV2^T^ and *Pb. terrae* COX were distinct; while both repressed CODH activity during exponential phase, *Pb. terrae* COX consumed CO regardless of the gas being added during growth, while *C. ulmosensis* CV2^T^ was far more conservative. This enhances our understanding of the potential controls of CO cycling by environmental factors and, crucially, increases the number of characterised carboxydovore isolates available in culture. Furthermore, the environmental significance of these strains, and of the CO-oxidising community, was considered. *C. ulmosensis* CV2^T^ was significantly more abundant in the youngest volcanic deposit, while *Pb. terrae* COX maintained low abundance in both tested deposits, suggesting that CO oxidation by the genus *Cupriavidus* may be greater than previously considered. On a community level, the Pseudomonadota were surprisingly of similar abundance between the two tested layers and there was little difference in overall community diversity. Many *coxL* OTUs were phylogenetically closely related to *Paraburkholderia* spp., corroborated by the dominance of *coxL* OTUs from this group within the Betaproteobacteria. In summary, this study furthers our understanding of not just the diversity of the carboxydovores colonising volcanic ecosystems of varying ages but also provides valuable physiological and metabolic context through which we can understand the survival of carboxydovore bacteria in extreme ecosystems.

Further genomic comparisons of the isolate CV2^T^ using MIGA, dDDH, ANI, and a set of 77 house-keeping genes using autoMLST, revealed similarities below the recommended ANI species-level thresholds (95% [104]) and DDH values (70% [105]) when the type strain genome sequences were used as query. Furthermore, *C. ulmosensis* CV2^T^ is unusual by possessing a form-I CODH and is the first member of this genus confirmed to oxidise CO. *C. ulmosensis* CV2^T^ is the representative of a novel species of the genus *Cupriavidus* within the order *Burkholderiales* (phylum *Pseudomonadota*). Based on this characterisation, we propose a new species, *C. ulmosensis* sp. nov.

### Description of *Cupriavidus ulmosensis* sp. nov

*C. ulmosensis* sp. nov. (ul.mo.sen’sis. N. L. masc n. *ulmosensis*, of the Parque Valle Los Ulmos, referring to Parque Valle Los Ulmos in Chile, park named after the ulmo tree in Calbuco Volcano, where the strain was isolated).

The type strain *C. ulmosensis* CV2^T^ (= NCIMB 15506^ T^, = CECT 30956^ T^), was isolated from soil of Calbuco Volcano in the Los Lagos Region, Chile. The genome is characterized by a size of 10.31 Mb and has a G + C content of 64.8 mol%. Gram-negative, rod-shaped aerobic heterotrophic bacterium, which produces copious extracellular polysaccharide visible under cryo-SEM (Figure S10). The isolate contains CODH-related genes and can grow in the presence of CO (100 – 100,000 ppmv CO). Cells grow at 25–37 °C at pH 5.0–8.0 (optimally at pH 7), tolerating 0–1% NaCl, and utilise a rage of non-sugar organic carbon sources in VL55 medium (pH 5.5).

## Supplementary Information

Additional file 1. 

## Data Availability

The genome sequences of the isolates have been deposited in the NCBI GenBank under the accession number PRJNA1001293 (SAMN36798297 for C. ulmosensis CV2T and SAMN36798260 for Pb. terrae COX). The 16S rRNA gene Sanger sequences have been deposited in NCBI under the accession numbers OR536588 for C. ulmosensis CV2T and OR536592 for Pb. terrae COX. The 16S rRNA gene and coxL gene sequencing data of the soils were deposited in the NCBI Sequence Read Archive (SRA) under the Bioproject accession numbers PRJNA1036796 and PRJNA1165783, respectively.

## Abbreviations

ANI: Average nucleotide identity
AAI: Average amino acid identity
ASV: Amplicon sequence variant
CO: Carbon monoxide
CODH: Carbon monoxide dehydrogenase
dDDH: Digital DNA–DNA hybridisation
MCD: Molybdopterin cytosine dinucleotide
OTU: Operational taxonomic unit
qPCR: Quantitative polymerase chain reaction
RA: Relative abundance
SRA: Sequence read archive
