# Supplementary material for: Carbon monoxide-oxidising Pseudomonadota on volcanic deposits
Source: Environ Microbiome. 2025 Jan 26;20:12. doi: 10.1186/s40793-025-00672-y (PMC11771112; doi:10.1186/s40793-025-00672-y)
Supplement: Supplementary file 1 — Additional file 1. [file 40793_2025_672_MOESM1_ESM.pdf]

Supplementary Information for

Carbon monoxide-oxidising Pseudomonadota on volcanic deposits.

Robin A. Dawson[1], Nicola Fantom[1], Tamara Martin-Pozas[2], Patricia Aguila[3], Gary M. King[4], Marcela Hernández[1]*

[1]School of Biological Sciences, University of East Anglia, Norwich, NR4 7TJ, UK

[2]Department of Biology and Geology, University of Almería, 04120 Almería, Spain

[3]Laboratorio de Microbiología Molecular, Escuela de Tecnología Médica, Universidad Austral de Chile, Juan Soler Manfredini 1771, Puerto Montt, Chile

[4]Department of Biological Sciences, Louisiana State University, LA 70803, USA

*Corresponding author: marcela.hernandez@uea.ac.uk

Running title:

CO oxidation by bacteria in volcanic soils

This supplement supplementary information includes:

- Supplementary methods
- Tables S1 and S13
- Figures S1 to S14
- Supplementary references

Supplementary methods

Physico-chemical analysis

Soil pH was measured by potentiometry in a soil/solution suspension ratio 1:2.5 H_2O. Phosphorus (P) was extracted by the Olsen bicarbonate method (pH 8.5). Organic matter was estimated by wet digestion with a modified Walkley-Black procedure analysed using an UV spectrophotometer (Unicam Helios E). For iron (Fe) and manganese (Mn) analyses, samples were dry-ashed at 500°C and diluted with HCl (2M) and the concentration was determined by atomic absorption spectrophotometry (AAS, Thermo Scientific iCE 3000 Series). Available sulfur (S) was extracted with $Ca(H_2PO_4)$ (20.01 mol/L) and analysed by turbidometry. Exchangeable cations (Ca, Mg, Na and K) were extracted with 1 m CH_3COONH_4 at pH 7.0 and analysed by AAS. Exchangeable Al was extracted with 1 M KCl and analysed by AAS, following the procedure described by Sadzawka *et al*. [1]. Other chemical analyses, including NH_4^+, were done by Kjeldahl method based on the wet combustion of the sample, by heating in sulfuric acid in the presence of a metallic catalyst, in order to reduce the organic nitrogen to ammonia, analysed by colorimetry. NO_3—N, were done using the colorimetric method with sulphosalicilic reaction. The Effective Cation Exchange Capacity (ECEC) is a measure of the total exchangeable cations a soil can hold at its natural pH. The methodology involves extracting and quantifying the exchangeable cations (Ca, Mg, K, Na, Al), measured as described above. Total N was measured using Dumas method [2] using Leco analyzer.

Table S1. Physicochemical features of Calbuco 2015 (tephra) layer and Calbuco 1917 (soil) layer samples.

	**2015**	**2015 Level**	**1917**	**1917 Level**
pH in Water	8.24	High	7.1	High
Organic Matter (%)	0.01	Low	7.05	Medium
Nitrates (NO_3-N) (mg/Kg)	0.1	Low	2	Low
Ammonium (NH_4-N) (mg/Kg)	2	Low	3.5	Low
Exchangeable Potassium (cmol/Kg)	0.04	Low	0.02	Low
Exchangeable Calcium (cmol/Kg)	0.17	Low	0.3	Low
Exchangeable Magnesium (cmol/Kg)	0.1	Low	0.05	Low
Exchangeable Sodium (cmol/Kg)	0.14	Low	0.06	Low
Exchangeable Aluminium (cmol/Kg)	0.01	Low	0.11	Low
Effective Cation Exchange Capacity (cmol/Kg)	0.46	Low	0.54	Low
Available Sulfur (mg/Kg)	1.4	Low	8.4	Low
Fe (mg/Kg)	28	High	50	High
Mn (mg/Kg)	6.4	High	1.2	Medium
Total N (%)	0.02		0.41	

Table S2. Growth substrate range of *C. ulmosensis* CV2^T and *Pb. terrae* COX. – indicates no growth; + indicates OD_{600} = 0.1-0.3; ++ indicates OD_{600} = 0.3-0.6; +++ indicates OD_{600} = 0.6-1.0 (n=2). Temperature and salinity growth profiles (n=3).

**Level of Growth**		
	***Cupriavidus ulmosensis* CV2**	***Paraburkholderia terrae* COX**
**Sugars**		
Glucose	-	++
Xylose	-	+++
Sucrose	-	-
Maltose	-	-
Lactose	-	+
L-Sorbose	-	-
L-Arabinose	+	++
Ribose	-	+++
**Sugar Alcohol**		
Sorbitol	-	++
**Carboxylic Acids**		
Succinate	++	++
Pyruvate	+++	++
Gluconate	++	+++
Salicylate	-	+
Phthalate	-	-
Potassium Citrate	++	+
Sodium Citrate	+++	+++
Methanesulfonate	-	-
Tartarate	+++	++
Methylmalonate	-	-
Propionate	-	-
Malate	++	++
Glyoxylate	++	+
Formate	-	-
Vanillate	-	-
Glutamate	+++	++
Taurine	+	+
**Temperature**		
25 °C	+++	++
30 °C	+++	++
37 °C	+	+
45 °C	-	-
**Salinity**		
0% NaCl	++	++
1% NaCl	++	++
10% NaCl	-	-

Table S3. Features of the genomes of *C. ulmosensis* CV2^T and *Paraburkholderia terrae* COX, determined by MicroScope and RAST annotation platforms.

Feature	*Cupriavidus ulmosensis* CV2	*Paraburkholderia terrae* COX
Size (bp)	10,313,057	10,305,210
GC Content (%)	64.8	62
Number of Contigs	651	256
L50	52	62
N50	61,988	101,247
Completeness (CheckM - %)	99.5	99.7
Contamination (CheckM - %)	2.3	2.4
No. of coding sequences (total)	10,380	10,390
No. of genes with assigned function	6,563	6041
No. of genes without assigned function	3,817	4349
No. of rRNAs	4	8
No. of tRNAs	63	53
No. of pseudogenes	12	8
**Subsystem**	***Cupriavidus ulmosensis* CV2**	***Paraburkholderia terrae* COX**
Cofactors, Vitamins, Prosthetic Groups, Pigments	303	268
Cell Wall and Capsule	39	46
Virulence, Disease and Defence	78	79
Potassium metabolism	22	17
Phages, Prophages, Transposable elements, Plasmids	8	7
Membrane Transport	137	98
Iron acquisition and metabolism	13	41
RNA Metabolism	75	65
Nucleosides and Nucleotides	106	118
Protein Metabolism	236	222
Cell Division and Cell Cycle	26	0
Motility and Chemotaxis	17	81
Regulation and Cell signalling	81	50
Secondary Metabolism	20	5
DNA Metabolism	119	101
Fatty Acids, Lipids, and Isoprenoids	279	216
Nitrogen Metabolism	29	28
Dormancy and Sporulation	1	1
Respiration	190	200
Stress Response	132	161
Metabolism of Aromatic Compounds	248	187
Amino Acids and Derivatives	769	622
Sulfur Metabolism	51	80
Phosphorus Metabolism	39	42
Carbohydrates	516	654

Table S4. Genes involved in amino acids biosynthesis on the genomes of *Cupriavidus* spp., identified through Comparative Analysis of MicroCyc metabolic pathways in MicroScope (https://mage.genoscope.cns.fr/microscope/metabolism/metabolicprofil.php).

Amino Acids Biosynthesis	Reactions nb	*Cupriavidus basilensis* OR16	*Cupriavidus campinensis* MJ1	***Cupriavidus ulmosensis* CV2^T**	*Cupriavidus metallidurans* BS1	*Cupriavidus necator* N-1^T	*Cupriavidus taiwanensis* LMG19424^T
alanine biosynthesis I	3	0.67	1	0.67	1	0.67	0.67
alanine biosynthesis II	1	1	1	1	1	0	0
alanine biosynthesis III	1	1	1	1	1	1	1
arginine biosynthesis I	9	0.89	1	1	1	1	0.89
arginine biosynthesis II (acetyl cycle)	9	0.89	0.89	1	1	1	0.89
arginine degradation I (arginase pathway)	3	0.33	0.67	0.33	0.33	0.33	0.33
asparagine biosynthesis I	1	1	0	1	0	1	0
asparagine biosynthesis II	1	0	0	0	0	1	0
asparagine biosynthesis III (tRNA-dependent)	3	0.67	0.67	0.67	0.67	0.67	0
aspartate biosynthesis	1	1	1	1	1	1	1
cysteine biosynthesis I	2	1	1	1	1	1	1
cysteine biosynthesis/homocysteine degradation	2	0	0	0.5	0.5	0.5	0.5
glutamate biosynthesis I	1	1	1	1	1	1	1
glutamate biosynthesis II	1	1	1	1	1	0	1
glutamate biosynthesis III	1	1	1	1	1	1	1
glutamate biosynthesis IV	1	0	1	0	1	0	0
glutamate biosynthesis V	1	0	1	0	1	1	0
glutamate degradation II	2	1	1	1	1	1	0.5
glutamine biosynthesis I	1	1	0	1	1	1	1
glycine biosynthesis I	1	1	1	1	1	1	1
glycine biosynthesis II	1	1	1	1	0	1	1
glycine biosynthesis III	1	0	1	0	1	0	1
glycine biosynthesis IV	1	1	1	1	1	1	1
histidine biosynthesis	10	1	0.9	1	0.9	0.9	1
homocysteine biosynthesis	2	0.5	1	1	1	1	1
homoserine biosynthesis	3	1	1	1	1	1	1
isoleucine biosynthesis I (from threonine)	5	1	0.8	1	1	1	1
isoleucine biosynthesis III	7	0.57	0.57	0.71	0.57	0.57	0.57
L-glutamine biosynthesis II (tRNA-dependent)	2	0.5	0.5	0.5	0.5	0.5	0
leucine biosynthesis	5	1	1	1	1	1	1
lysine biosynthesis I	9	1	1	1	0.89	0.89	0.89
lysine biosynthesis VI	7	0.86	0.86	0.86	0.71	1	0.86
methionine biosynthesis I	5	0.6	0.6	0.6	0.8	0.8	0.6
ornithine biosynthesis	5	0.8	1	1	1	1	1
phenylalanine biosynthesis I	3	1	1	1	0.67	1	1
phenylalanine biosynthesis II	3	0.67	0.33	0.67	0	1	0.33
proline biosynthesis I	3	1	1	1	1	1	1
S-adenosyl-L-methionine cycle I	5	0.6	0.6	0.6	0.6	0.6	0.6
S-adenosyl-L-methionine cycle II	4	0.75	0.75	0.75	0.75	0.75	0.75
selenocysteine biosynthesis I (bacteria)	3	0.67	0.67	0.67	0.67	0.67	1
serine biosynthesis	3	1	1	1	1	1	1
threonine biosynthesis from homoserine	2	1	1	1	1	1	1
tryptophan biosynthesis	6	1	0.83	1	1	1	1
tyrosine biosynthesis I	3	1	1	1	0.67	1	1
tyrosine biosynthesis III	3	1	0.33	0.67	0	0.67	0.67
tyrosine biosynthesis IV	1	1	0	1	1	1	1
uracil degradation II (reductive)	3	0	0.33	0.33	0.67	0.67	0
valine biosynthesis	4	1	1	1	1	1	1
β-alanine biosynthesis II	6	0.67	0.33	0.67	0.33	0.5	0.33
β-alanine biosynthesis III	1	1	1	1	0	1	1

Table S5. Genes involved in carbohydrates biosynthesis on the genomes of *Cupriavidus* spp., identified through Comparative Analysis of MicroCyc metabolic pathways in MicroScope (https://mage.genoscope.cns.fr/microscope/metabolism/metabolicprofil.php).

Carbohydrates Biosynthesis	Reactions nb	*Cupriavidus basilensis* OR16	*Cupriavidus campinensis* MJ1	***Cupriavidus ulmosensis* CV2**[T]	*Cupriavidus metallidurans* BS1	*Cupriavidus necator* N-1[T]	*Cupriavidus taiwanensis* LMG19424[T]
2-keto-L-gulonate biosynthesis	3	0	0	0.33	0	0	0
acetaldehyde biosynthesis I	2	**1**	0.5	1	0.5	0.5	0.5
acetaldehyde biosynthesis II	1	**1**	0	1	0	0	0
ADP-L-glycero-β-D-manno-heptose biosynthesis	5	0.6	0.8	1	**1**	0.8	0.8
Calvin-Benson-Bassham cycle	13	0.62	0.62	0.62	0.69	0.85	0.85
CDP-3,6-dideoxyhexose biosynthesis	3	0.67	**1**	1	0.33	0	0.67
cellulose biosynthesis	1	**1**	**1**	1	**1**	**1**	1
CMP-KDO biosynthesis I	4	0.75	0.75	0.75	0.75	0.75	0.75
CMP-N-acetylneuraminate biosynthesis II (bacteria)	3	0	0	0.33	0.33	0.33	0
CMP-pseudaminate biosynthesis	6	0.17	0	0	0	0	0
dTDP-3-acetamido-3,6-dideoxy-α-D-galactose biosynthesis	5	0.4	0.4	1	0.4	0.4	0.4
dTDP-D-desosamine biosynthesis	6	0.33	0.33	0.33	0.5	0.33	0.33
dTDP-L-rhamnose biosynthesis I	4	**1**	**1**	1	**1**	**1**	1
GDP-L-fucose biosynthesis I (from GDP-D-mannose)	2	**1**	**1**	1	**1**	**1**	1
GDP-mannose biosynthesis	4	0.75	**1**	0.75	**1**	**1**	1
gluconeogenesis I	13	0.77	0.77	0.92	0.77	0.92	0.92
gluconeogenesis II (Methanobacterium thermoautotrophicum)	20	0.65	0.55	0.65	0.65	0.75	0.65
glycogen biosynthesis I (from ADP-D-Glucose)	3	0.33	0.67	0.67	0	0.33	0.33
glycogen degradation I	7	0.57	0.71	0.71	0.29	0.57	0.57
itaconate biosynthesis	4	0.75	0.5	1	0.5	0.75	0.75
UDP-D-xylose and UDP-D-glucuronate biosynthesis	2	0.5	**1**	0.5	**1**	0.5	1

Table S6. Genes involved in Cell Structures biosynthesis on the genomes of *Cupriavidus* spp., identified through Comparative Analysis of MicroCyc metabolic pathways in MicroScope (https://mage.genoscope.cns.fr/microscope/metabolism/metabolicprofil.php).

Cell structures biosynthesis	Reactions nb	*Cupriavidus basilensis* OR16	*Cupriavidus campinensis* MJ1	***Cupriavidus ulmosensis*** **CV2**[T]	*Cupriavidus metallidurans* BS1	*Cupriavidus necator* N-1[T]	*Cupriavidus taiwanensis* LMG19424[T]
CDP-3,6-dideoxyhexose biosynthesis	3	0.67	**1**	1	0.33	0	0.67
cellulose biosynthesis	1	**1**	**1**	1	**1**	**1**	1
colanic acid building blocks biosynthesis	11	0.73	0.82	0.73	0.82	0.82	0.82
dTDP-L-rhamnose biosynthesis I	4	**1**	**1**	1	**1**	**1**	1
enterobacterial common antigen biosynthesis	9	0.22	0.33	0.44	0.33	0.44	0.22
KDO transfer to lipid IVA I	2	0.5	0.5	0.5	**1**	**1**	1
Kdo transfer to lipid IVA II (Chlamydia)	5	0.2	0.2	0.2	**1**	0.4	0.4
lipid IVA biosynthesis	6	**1**	0.83	1	**1**	**1**	1
O-antigen building blocks biosynthesis (E. coli)	9	0.89	0.89	0.89	0.89	0.89	0.89
peptidoglycan biosynthesis I (meso-diaminopimelate containing)	13	0.77	**1**	1	**1**	0.92	1
peptidoglycan biosynthesis III (mycobacteria)	14	0.57	0.71	0.71	0.86	0.57	0.71
polymyxin resistance	6	0.33	0.67	0.5	0.83	0.5	0.33
superpathway of (KDO)2-lipid A biosynthesis	16	0.69	0.63	0.63	0.75	0.75	0.81
UDP-N-acetyl-D-glucosamine biosynthesis I	4	**1**	**1**	1	**1**	**1**	1
UDP-N-acetylmuramoyl-pentapeptide biosynthesis III (meso-DAP-containing)	8	0.75	**1**	1	**1**	**1**	1

Table S7. Genes involved in Fatty Acids and Lipids biosynthesis on the genomes of *Cupriavidus* spp., identified through Comparative Analysis of MicroCyc metabolic pathways in MicroScope (https://mage.genoscope.cns.fr/microscope/metabolism/metabolicprofil.php).

Fatty acids and lipids biosynthesis	Reactions nb	*Cupriavidus basilensis* OR16	*Cupriavidus campinensis* MJ1	***Cupriavidus ulmosensis* CV2**^{T}	*Cupriavidus metallidurans* BS1	*Cupriavidus necator* N-1^{T}	*Cupriavidus taiwanensis* LMG19424^{T}
(KDO)2-lipid A biosynthesis I	2	0.5	0.5	0	0.5	0.5	1
acyl-CoA hydrolysis	1	**1**	**1**	1	**1**	**1**	1
biotin-carboxyl carrier protein assembly	3	**1**	**1**	1	**1**	**1**	1
cardiolipin biosynthesis I	3	**1**	**1**	1	**1**	**1**	1
cardiolipin biosynthesis II	3	**1**	**1**	1	**1**	**1**	1
CDP-diacylglycerol biosynthesis I	4	**1**	0.75	1	0.75	**1**	0.75
CDP-diacylglycerol biosynthesis II	4	**1**	0.75	1	0.75	**1**	0.75
CDP-diacylglycerol biosynthesis III	5	0.8	0.4	0.8	0.8	0.8	0.6
cis-dodecenoyl biosynthesis	6	0.83	0.67	0.83	0.67	0.83	0.5
cis-vaccenate biosynthesis	5	0.4	0.6	0.6	0.6	0.6	0.6
cyclopropane fatty acid (CFA) biosynthesis	1	**1**	**1**	0	**1**	**1**	1
fatty acid activation	1	**1**	**1**	1	**1**	**1**	1
fatty acid biosynthesis initiation I	3	**1**	**1**	1	**1**	**1**	1
fatty acid biosynthesis initiation II	2	**1**	0.5	1	0.5	**1**	1
fatty acid elongation -- saturated	5	**1**	**1**	1	**1**	**1**	0.8
fatty acids biosynthesis (yeast)	1	0	0	1	0	**1**	0
KDO transfer to lipid IVA I	2	0.5	0.5	0.5	**1**	**1**	1
Kdo transfer to lipid IVA II (Chlamydia)	5	0.2	0.2	0.2	**1**	0.4	0.4
Lipid A-core biosynthesis	10	0.2	0.1	0.2	0.2	0.1	0.1
lipid IVA biosynthesis	6	**1**	0.83	1	**1**	**1**	1
mevalonate pathway I	7	0.29	0.14	0.29	0.14	0.14	0.14
palmitate biosynthesis II (bacteria and plants)	29	0.93	0.93	0.93	0.93	0.93	0.93
palmitoleate biosynthesis I	9	**1**	0.78	1	0.89	0.78	0.78
phosphatidylcholine biosynthesis I	3	0.33	0.33	0.33	0.33	0	0
phosphatidylethanolamine biosynthesis I	2	**1**	**1**	1	**1**	**1**	1
phosphatidylglycerol biosynthesis I (plastidic)	6	**1**	0.83	1	0.83	**1**	0.83
phosphatidylglycerol biosynthesis II (non-plastidic)	6	**1**	0.83	1	0.83	**1**	0.83
stearate biosynthesis II (plants)	5	0.8	0.6	0.8	0.8	0.6	0.8
superpathway of (KDO)2-lipid A biosynthesis	16	0.69	0.63	0.63	0.75	0.75	0.81

Table S8. Genes involved in Secondary Metabolites biosynthesis on the genomes of *Cupriavidus* spp., identified through Comparative Analysis of MicroCyc metabolic pathways in MicroScope (https://mage.genoscope.cns.fr/microscope/metabolism/metabolicprofil.php).

Secondary metabolites biosynthesis	Reactions nb	*Cupriavidus basilensis* OR16	*Cupriavidus campinensis* MJ1	***Cupriavidus ulmosensis* CV2**[T]	*Cupriavidus metallidurans* BS1	*Cupriavidus necator* N-1[T]	*Cupriavidus taiwanensis* LMG19424[T]
acetaldehyde biosynthesis II	1	**1**	0	1	0	0	0
betaxanthin biosynthesis (via dopamine)	1	0	0	1	0	0	0
camptothecin biosynthesis	5	0	0	0.2	0	0	0
epoxysqualene biosynthesis	3	0.67	0.67	0.67	0.67	0.67	0.67
fluoroacetate and fluorothreonine biosynthesis	6	0	0.33	0.33	0.33	0	0
isopenicillin N biosynthesis	2	0	0	0	0	0	0.5
mevalonate pathway I	7	0.29	0.14	0.29	0.14	0.14	0.14
myo-inositol biosynthesis	2	0.5	0.5	0.5	0.5	0	0
neurosporene biosynthesis	5	0.4	0.4	0.4	0	0.4	0.4
phenazine-1-carboxylate biosynthesis	5	0	0.2	0.2	0	0.2	0
phenylethanol biosynthesis	4	0.25	0.25	0.5	0.25	0.25	0.25
preQ0 biosynthesis	4	0.75	0.75	0.75	0.75	0.75	0.5
pyrrolnitrin biosynthesis	4	0	0.25	0	0	0	0
trans-lycopene biosynthesis I (bacteria)	6	0.33	0.33	0.33	0	0.33	0.33
tryptophan degradation VI (via tryptamine)	3	0	0	0.33	0.33	0	0

Table S9. Genes involved in C1 Compounds Utilisation on the genomes of *Cupriavidus* spp., identified through Comparative Analysis of MicroCyc metabolic pathways in MicroScope (https://mage.genoscope.cns.fr/microscope/metabolism/metabolicprofil.php).

C1 Compounds Utilization and Assimilation	Reactions nb	*Cupriavidus basilensis* OR16	*Cupriavidus campinensis* MJ1	***Cupriavidus ulmosensis*** **CV2[T]**	*Cupriavidus metallidurans* BS1	*Cupriavidus necator* N-1[T]	*Cupriavidus taiwanensis* LMG19424[T]
Calvin-Benson-Bassham cycle	13	0.62	0.62	0.62	0.69	0.85	0.85
CO2 fixation into oxaloacetate (anapleurotic)	2	**1**	**1**	1	**1**	**1**	1
formaldehyde assimilation I (serine pathway)	12	0.75	0.75	0.75	0.75	0.83	0.67
formaldehyde oxidation II (glutathione-dependent)	2	**1**	**1**	1	**1**	**1**	0.5
formaldehyde oxidation IV (thiol-independent)	1	0	0	0	**1**	0	0
formaldehyde oxidation V (tetrahydrofolate pathway)	3	0.67	0.67	0.67	0.67	0.67	0.67
formate oxidation to CO2	1	**1**	**1**	1	**1**	**1**	1
methane oxidation to methanol I	1	0	0	0	0	**1**	0
methanol oxidation to formaldehyde I	1	0	0	0	0	0	1
methanol oxidation to formaldehyde II	1	0	0	0	0	**1**	0
reductive acetyl coenzyme A pathway	8	0.5	0.38	0.5	0.63	0.75	0.5

Table S10. Genes involved in Carbohydrates degradation on the genomes of *Cupriavidus* spp., identified through Comparative Analysis of MicroCyc metabolic pathways in MicroScope (https://mage.genoscope.cns.fr/microscope/metabolism/metabolicprofil.php).

Carbohydrates Degradation	Reactions nb	*Cupriavidus basilensis* OR16	*Cupriavidus campinensis* MJ1	***Cupriavidus ulmosensis* CV2**^{T}	*Cupriavidus metallidurans* BS1	*Cupriavidus necator* N-1^{T}	*Cupriavidus taiwanensis* LMG19424^{T}
acetoin degradation	2	0.5	0.5	1	0.5	0.5	0.5
cellulose degradation I (cellulosome)	8	0.13	0.13	0.13	0.13	0.25	0.13
chondroitin sulfate and dermatan sulfate degradation I (bacterial)	13	0	0	0.08	0	0	0
D-mannose degradation	1	**1**	**1**	1	**1**	**1**	1
galactose degradation I (Leloir pathway)	5	0.4	0.4	0.4	0.4	0.4	0.4
glucose and glucose-1-phosphate degradation	5	0.8	0.6	0.8	0.4	0.4	0.6
glucose degradation (oxidative)	5	0.8	0.2	0.6	0.2	0.6	0.6
glycogen degradation I	7	0.57	0.71	0.71	0.29	0.57	0.57
glycogen degradation II	5	0.4	0.8	0.6	0.2	0.6	0.4
glycogen degradation III	6	0.5	0.67	0.67	0.17	0.67	0.33
L-arabinose degradation III	5	0.4	0.2	0.6	0.2	0.4	0
L-rhamnose degradation II	8	0.5	0.13	0.63	0	0.75	0.13
superpathway of cellulose and hemicellulose degradation (cellulolosome)	11	0.09	0.09	0.18	0.18	0.18	0.18
trehalose degradation II (trehalase)	2	**1**	**1**	1	0.5	**1**	1
xylose degradation I	2	0	0	0	0	0.5	0
xylose degradation IV	17	0.88	0.71	0.82	0.71	0.94	0.82

Table S11. Genes involved in Generation of Precursor Metabolites on the genomes of *Cupriavidus* spp., identified through Comparative Analysis of MicroCyc metabolic pathways in MicroScope (https://mage.genoscope.cns.fr/microscope/metabolism/metabolicprofil.php).

Generation of Precursor Metabolites	Reactions nb	*Cupriavidus basilensis* OR16	*Cupriavidus campinensis* MJ1	***Cupriavidus ulmosensis*** **CV2[T]**	*Cupriavidus metallidurans* BS1	*Cupriavidus necator* N-1[T]	*Cupriavidus taiwanensis* LMG19424[T]
ammonia oxidation I (aerobic)	2	0.5	**1**	0.5	0	0.5	0.5
formate oxidation to CO2	1	**1**	**1**	1	**1**	**1**	1
hydrogen oxidation I (aerobic)	1	0	0	1	0	0	1
hydrogen oxidation II (aerobic, NAD)	1	0	0	1	0	0	1
hydrogen oxidation III (anaerobic, NADP)	1	**1**	0	1	0	0	0
sulfide oxidation I (sulfide-quinone reductase)	1	0	0	1	**1**	0	0
sulfide oxidation III (sulfur dioxygenase)	1	0	**1**	1	**1**	**1**	0
sulfite oxidation I (sulfite oxidoreductase)	1	**1**	0	1	**1**	**1**	1
sulfur oxidation I (aerobic)	1	0	**1**	1	**1**	**1**	0

Table S12. Alpha-diversity indices calculated using 16S rRNA gene amplicon sequencing data for triplicate soil/sediment DNA extracts.

	Observed	Shannon	Simpson	Evenness
Calbuco_2015.1	1414	5.53	0.99	0.76
Calbuco_2015.2	1462	5.15	0.97	0.71
Calbuco_2015.3	1430	5.41	0.98	0.74
Calbuco_1917.1	1501	5.42	0.99	0.74
Calbuco_1917.2	1490	5.40	0.98	0.74
Calbuco_1917.3	1097	4.52	0.95	0.65

Table S13. Top 10 results of BLASTp analysis of CoxL from *C. ulmosensis* CV2^T against the NCBI database (nr).

Description	Scientific Name	Max Score	Total Score	Query Cover	E value	Per. ident	Acc. Len	Accession
aerobic carbon-monoxide dehydrogenase large subunit [*Cupriavidus* sp. CV2]	*Cupriavidus* sp. CV2	1652	1652	99%	0	100	800	WP_318231694.1
aerobic carbon-monoxide dehydrogenase large subunit [*Cupriavidus* sp. CuC1]	*Cupriavidus* sp. CuC1	1646	1646	99%	0	99.5	800	WP_400100062.1
aerobic carbon-monoxide dehydrogenase large subunit [*Cupriavidus basilensis*]	*Cupriavidus basilensis*	1630	1630	100%	0	98.13	803	WP_174427970.1
aerobic carbon-monoxide dehydrogenase large subunit [*Cupriavidus* sp. SK-3]	*Cupriavidus* sp. SK-3	1624	1624	100%	0	97.14	803	WP_035876025.1
aerobic carbon-monoxide dehydrogenase large subunit [*Cupriavidus basilensis*]	*Cupriavidus basilensis*	1623	1623	100%	0	97.01	804	MDF3881665.1
aerobic carbon-monoxide dehydrogenase large subunit [*Cupriavidus basilensis*]	*Cupriavidus basilensis*	1622	1622	100%	0	97.01	803	WP_346430134.1
carbon monoxide dehydrogenase [Burkholderiaceae bacterium 16]	Burkholderiaceae bacterium 16	1617	1617	100%	0	96.89	803	KJK25661.1
aerobic carbon-monoxide dehydrogenase large subunit [*Cupriavidus basilensis*]	*Cupriavidus basilensis*	1533	1533	100%	0	94.52	803	WP_059411061.1
aerobic carbon-monoxide dehydrogenase large subunit [*Paraburkholderia* sp. BL10I2N1]	*Paraburkholderia* sp. BL10I2N1	1531	1531	99%	0	92.62	803	WP_133663049.1
TPA: aerobic carbon-monoxide dehydrogenase large subunit [*Paraburkholderia* sp.]	*Paraburkholderia* sp.	1527	1527	99%	0	91	804	HEY4805849.1

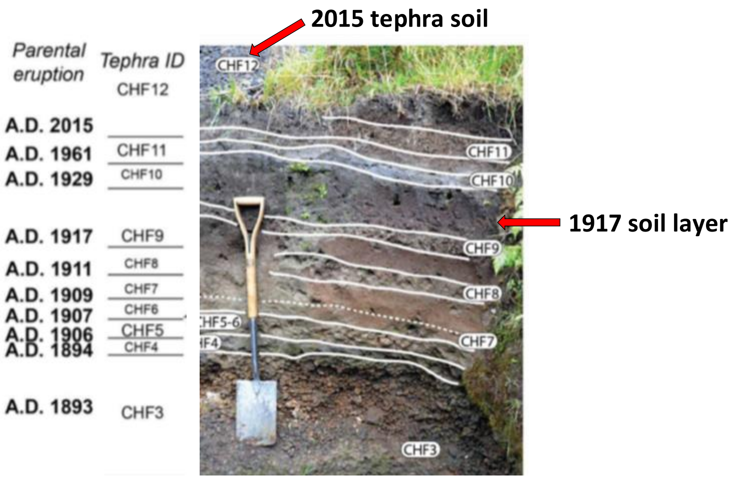

Figure S1. Calbuco volcano stratification formed by successive pyroclastic deposits. Sampling locations (CHF12 and CHF9) were deposited during eruptions in 2015 and 1917. Figure adapted from Romero *et al*. [3].

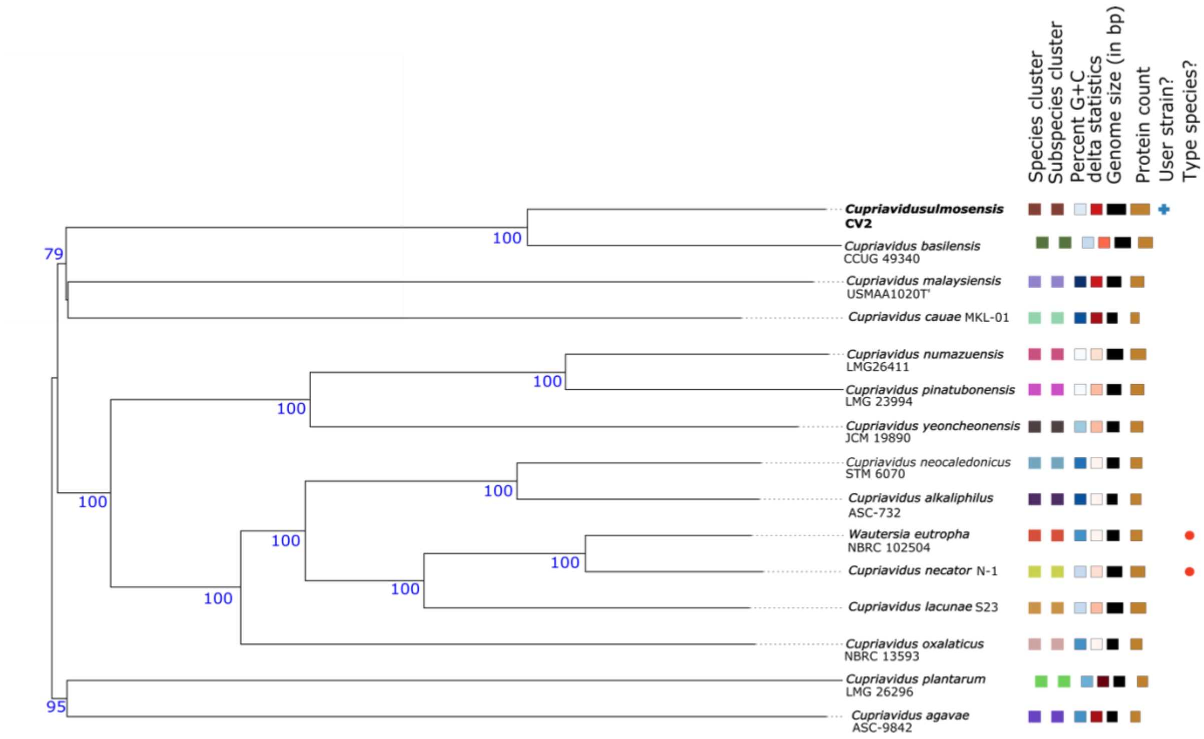

Figure S2. Whole genome-based taxonomic analysis of *C. ulmosensis* CV2^{T} against representative genomes of *Cupriavidus* spp., generated using TYGS (Type Strain Genome Server). Branch support was inferred from 100 pseudo-bootstrap replicates each.

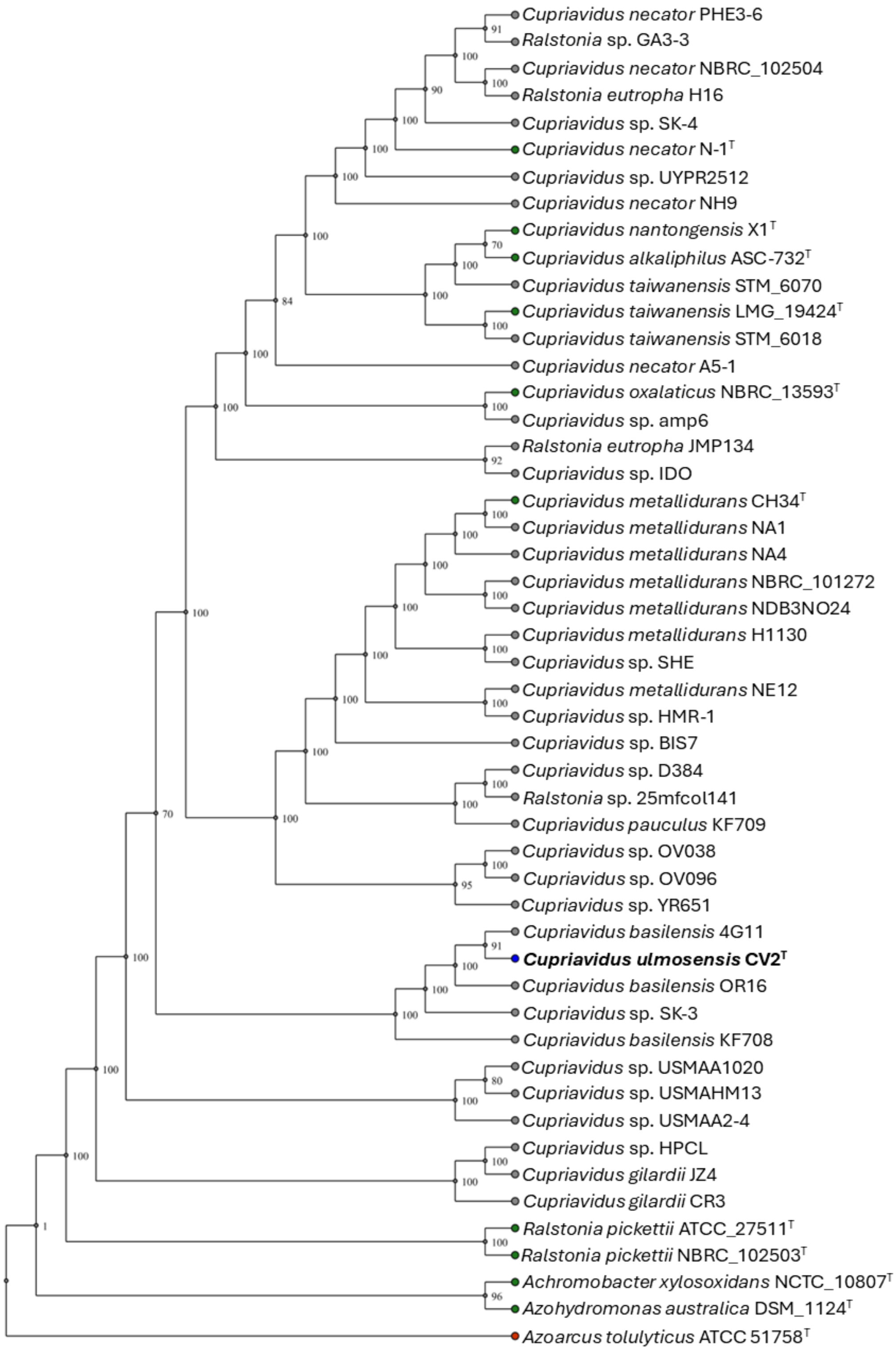

Figure S3. Multi-locus species tree analysis (autoMLST) of reference *Cupriavidus* spp. genomes against *C. ulmosensis* CV2^{T}. Bootstrap values were generated using 1000 replications.

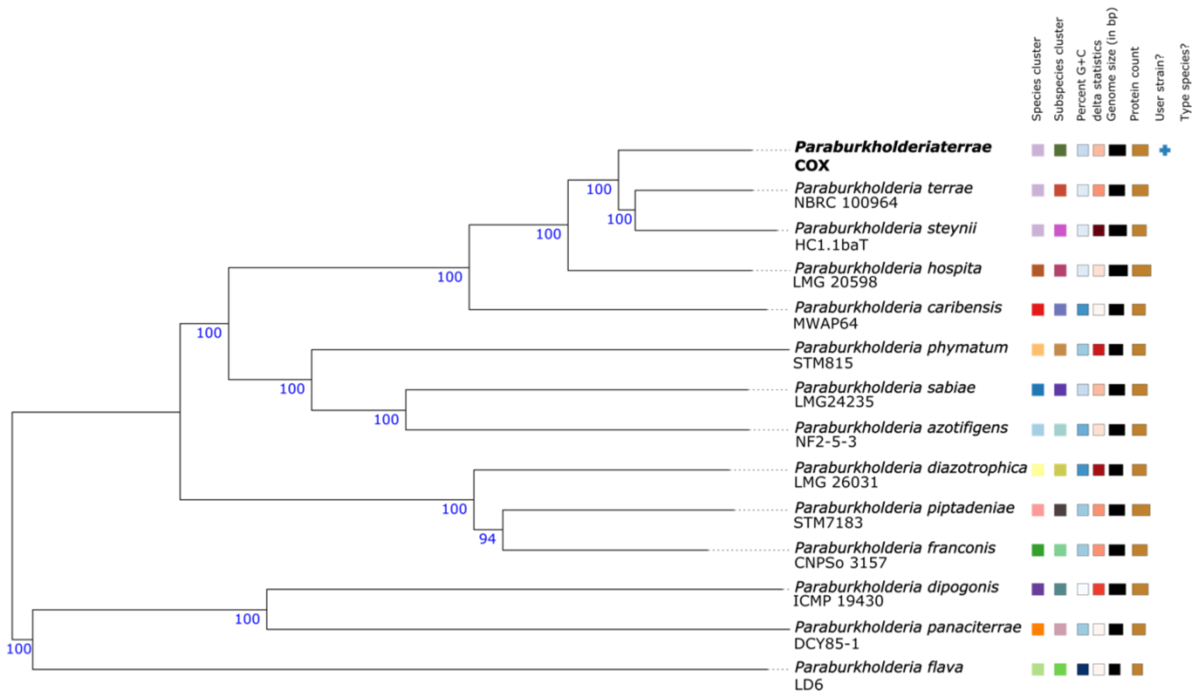

Figure S4. Whole genome-based taxonomic analysis of *Pb. terrae* COX against representative genomes of *Paraburkholderia* spp., generated using TYGS (Type Strain Genome Server). Branch support was inferred from 100 pseudo-bootstrap replicates each.

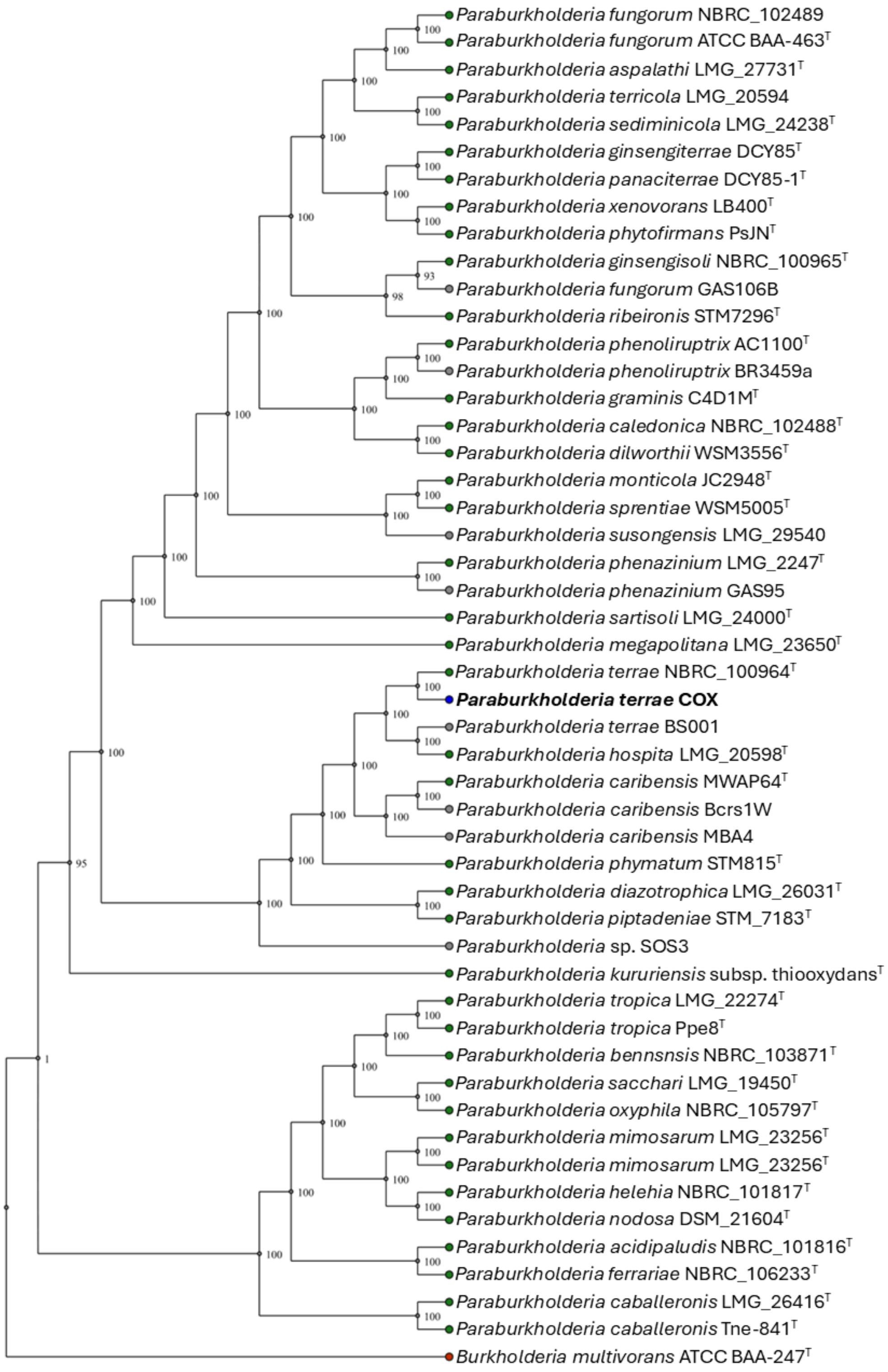

Figure S5. Multi-locus species tree analysis (autoMLST) of reference *Paraburkholderia* spp. genomes against *Pb. terrae* COX. Bootstrap values were generated using 1000 replications.

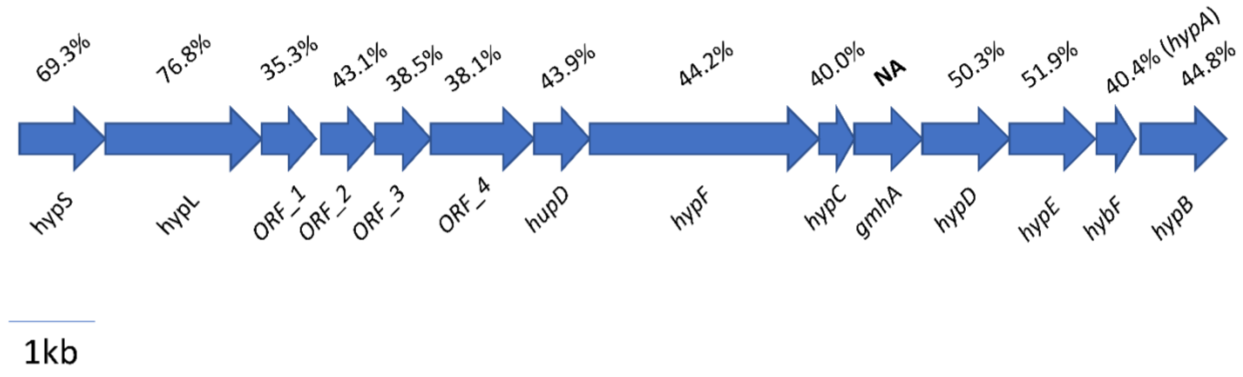

Figure S6. Gene cluster encoding a putative Ni-Fe hydrogenase in *C. ulmosensis* CV2^T. Identity of translated amino acids (%) were calculated relative to the hydrogenase gene cluster in *C. necator* H16.

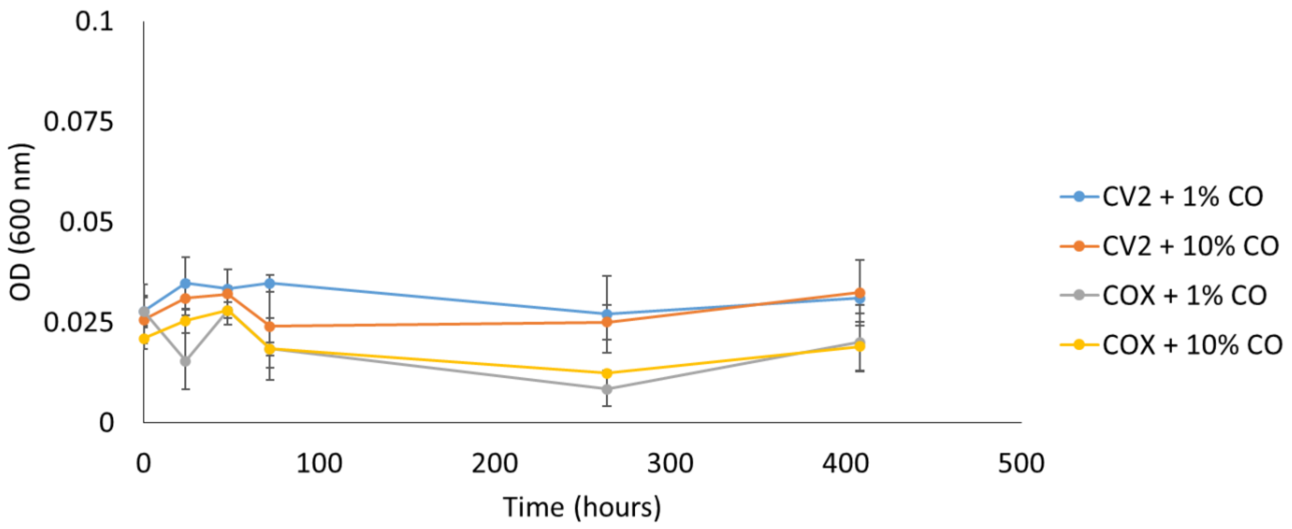

Figure S7. Confirmation of carboxydovory by incubation of *C. ulmosensis* CV2^T and *Paraburkholderia terrae* COX with 1% (v/v) or 10% (v/v) CO as the sole source of carbon and energy (n=3). Error bars represent the standard deviation about the mean.

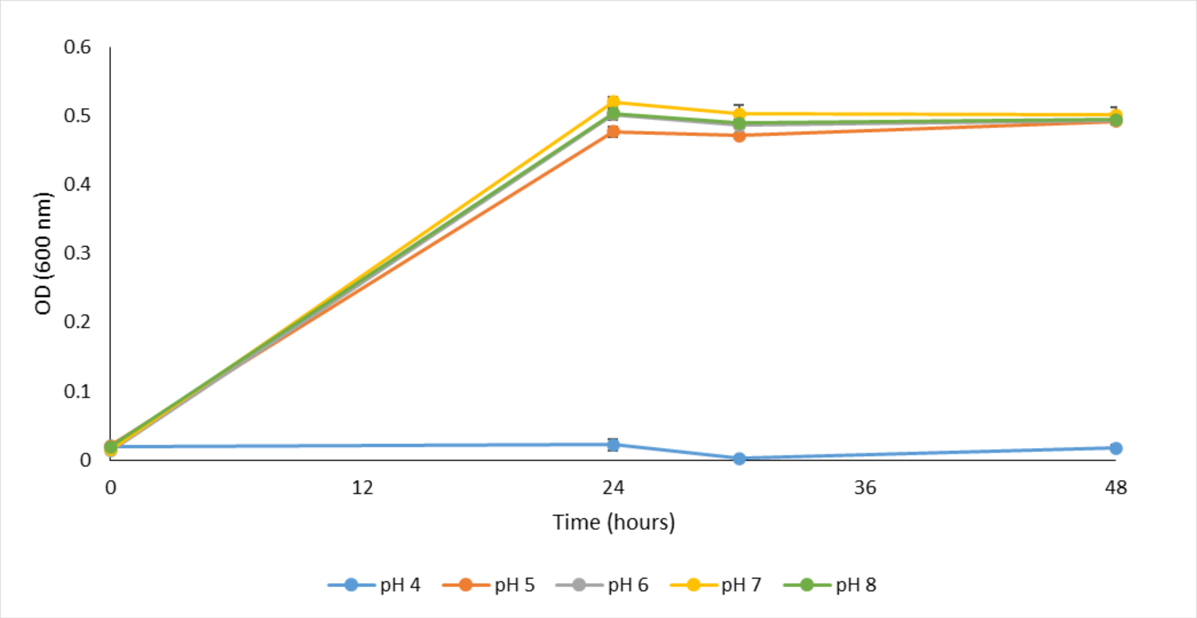

Figure S8. Growth of *C. ulmosensis* CV2^T on 5 mM pyruvate in VL55 medium adjusted to pH 4, 5, 6, 7 or 8 using HCl and NaOH (n=3). Error bars represent the standard deviation about the mean. Average final pH values were as follows for pH 4.0 (4.1), pH 5.0 (6.0), pH 6.0 (6.60), pH 7.0 (7.60), pH 8.0 (7.70).

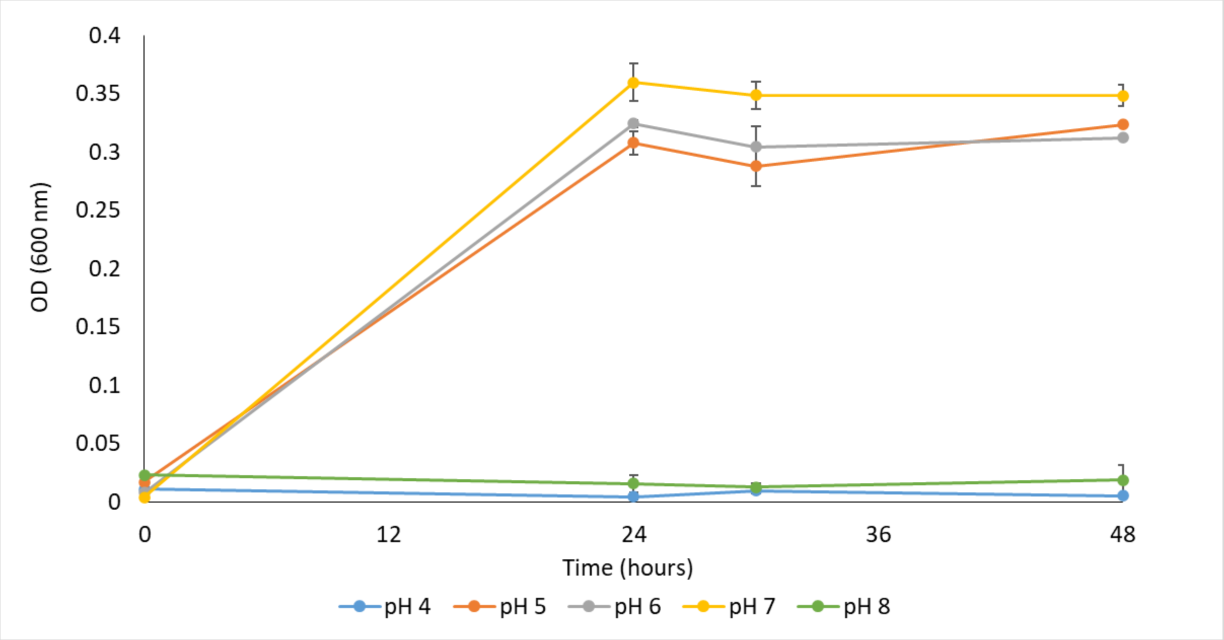

Figure S9. Growth of *Pb. terrae* COX on 5 mM pyruvate in VL55 medium adjusted to pH 4, 5, 6, 7 or 8 using HCl and NaOH (n=3). Error bars represent the standard deviation about the mean. Average final pH values were as follows for pH 4.0 (4.1), pH 5.0 (6.0), pH 6.0 (6.50), pH 7.0 (7.40), pH 8.0 (7.60).

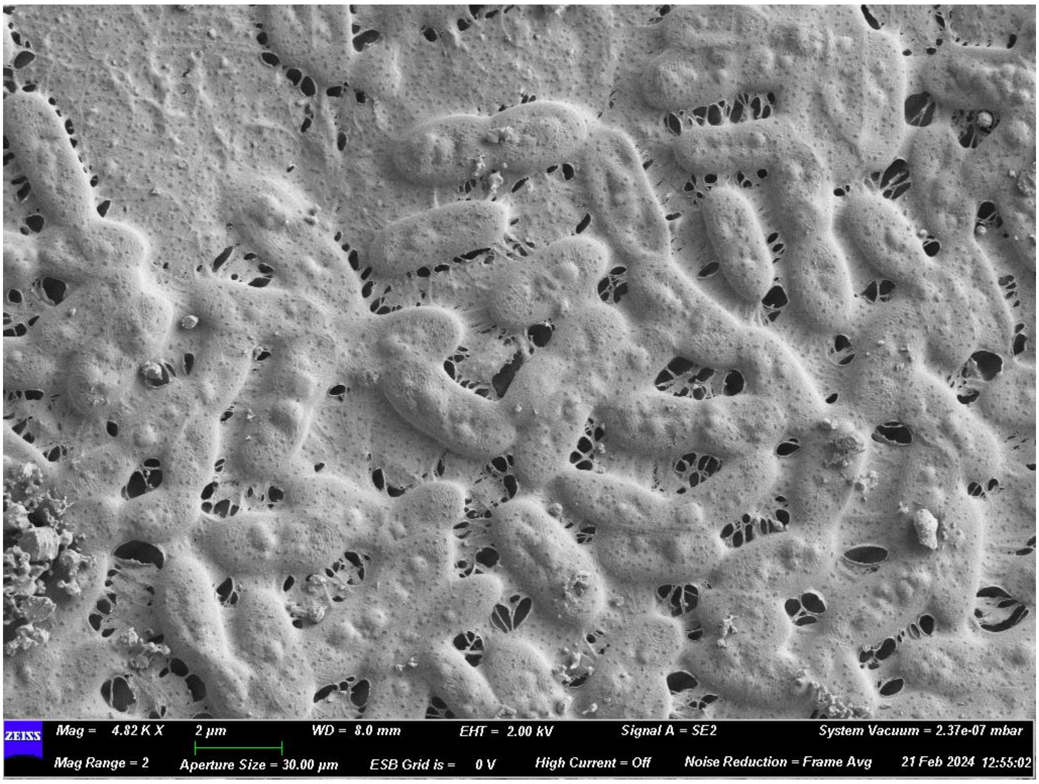

Figure S10. Scanning electron micrograph of *C. ulmosensis* CV2^{T}. Image settings are displayed in the technical panel.

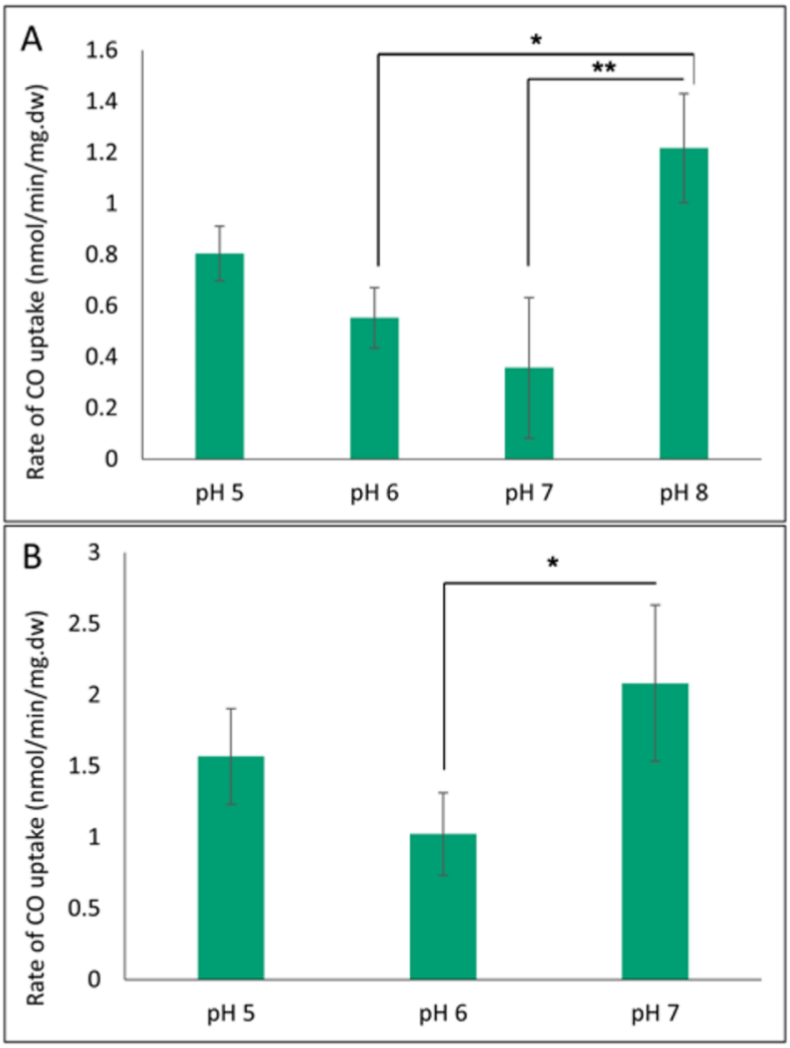

Figure S11. Rate of CO oxidation by (A) *C. ulmosensis* CV2[T] and (B) *P. terrae* COX after growth at pH 5, 6, 7, or 8. Statistical significance between conditions is indicated by an asterisk (* p≤0.05; **p≤0.01). Error bars represent the standard deviation about the mean.

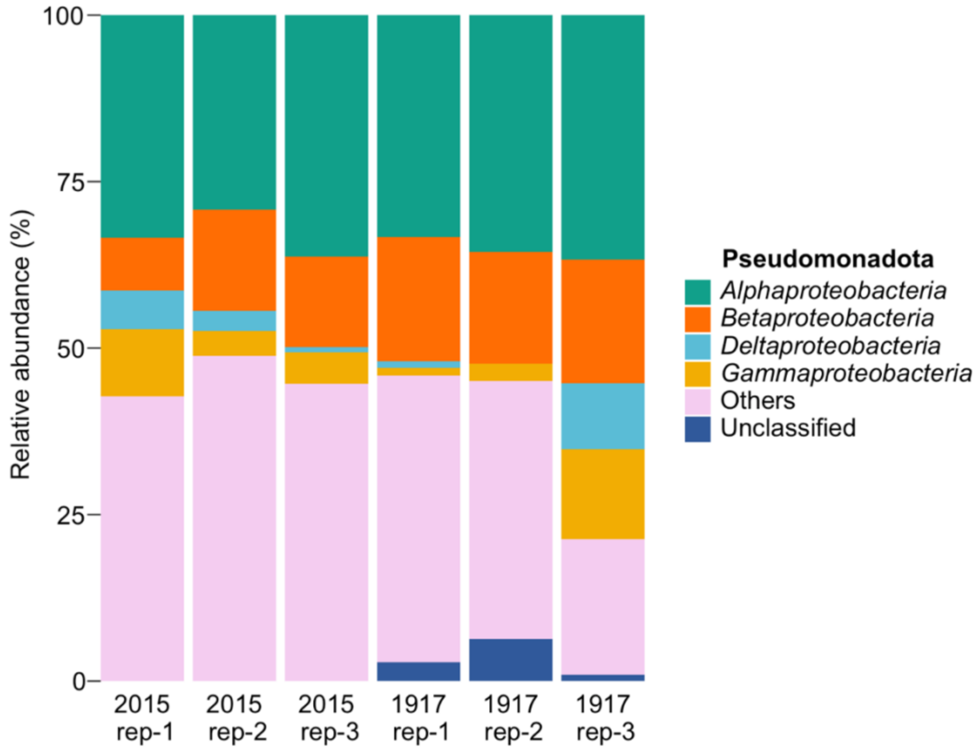

Figure S12. Relative abundances of 16S rRNA gene sequences from the phylum Pseudomonadota, identified in tephra layers from 2015 and 1917 eruptions.

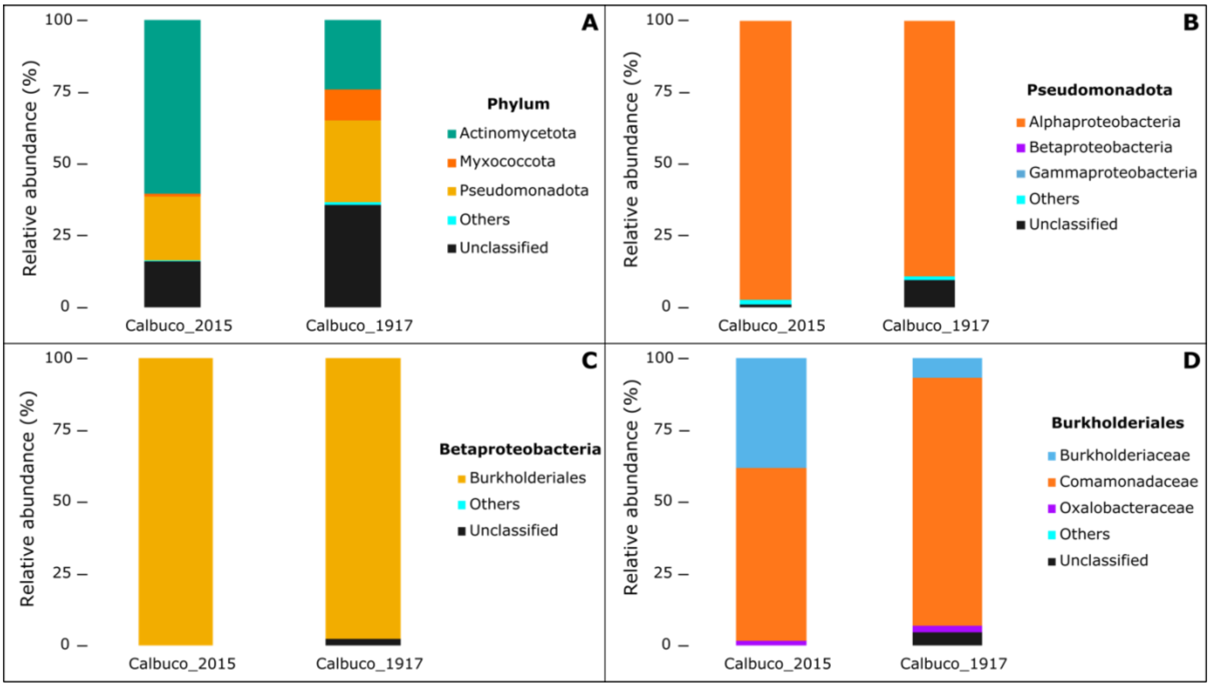

Figure S13. Relative abundances of OTUs related to *coxL* from different A) phyla, B) classes of the phylum Pseudomonadota, C) orders of the class Betaproteobacteria or D) families of the order Burkholderiales, identified in tephra layers from 2015 and 1917 eruptions.

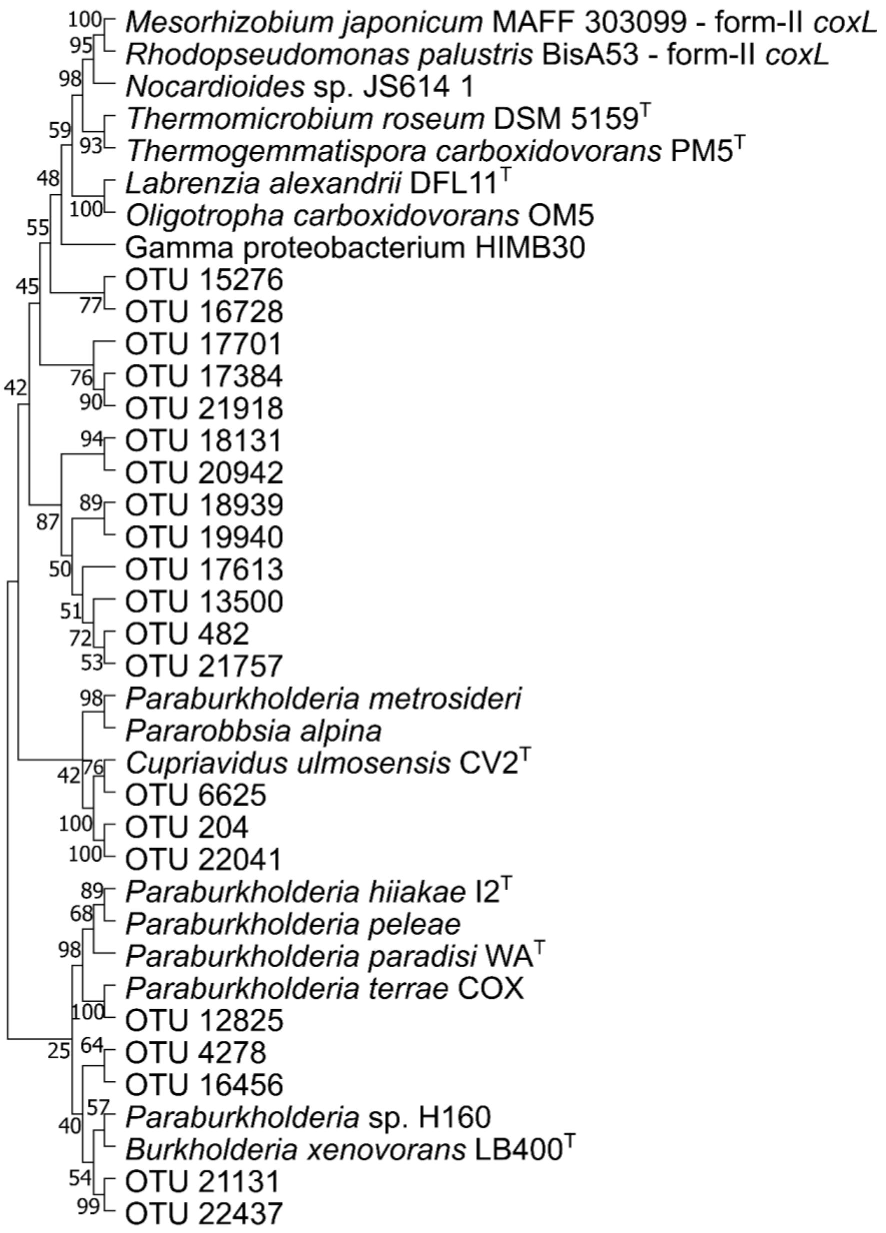

Figure S14. Evolutionary relatedness of translated form-I CoxL amino acid sequences derived from *coxL* OTUs from Calbuco volcano. *C. ulmosensis* CV2^{T} and *Pb. terrae* COX were used as references along with published CoxL sequences from known CO oxidising bacteria, and form-II CoxL amino acids were used as an outgroup. The tree was drawn using the Maximum Likelihood method with 500 Bootstrap replicates in MEGA11.

Supplementary References

1. Sadzawka A, Carrasco M, Grez RZ, De La Luz Mora M, Flores HP, Neaman A. Metodos recomendados para los suelos de Chile. Santiago; 2006.

2. Saint-Denis T, Goupy J. Optimization of a nitrogen analyser based on the Dumas method. Anal Chim Acta. 2004;515:191–8.

3. Romero JE, Alloway B V., Gutiérrez R, Bertín D, Castruccio A, Villarosa G, et al. Centennial-scale eruptive diversity at Volcán Calbuco (41.3°S; Northwest Patagonia) deduced from historic tephra cover-bed and dendrochronologic archives. J Volcanol Geotherm Res. 2021;417.
